# β-catenin activation down-regulates cell-cell junction-related genes and induces epithelial-to-mesenchymal transition in colorectal cancers

**DOI:** 10.1038/s41598-019-54890-9

**Published:** 2019-12-05

**Authors:** Won Kyu Kim, Yujin Kwon, Mi Jang, Minhee Park, Jiyoon Kim, Suyeon Cho, Dong Geon Jang, Wook-Bin Lee, Sang Hoon Jung, Hye Jin Choi, Byung Soh Min, Tae Il Kim, Sung Pil Hong, Young-Ki Paik, Hoguen Kim

**Affiliations:** 1 Brain Korea 21 PLUS Project for Medical Science, Seoul, Korea; 2 Departments of Pathology, Yonsei University College of Medicine, Seoul, 120-752 Korea; 3 Natural Product Research Center, Korea Institute of Science and Technology (KIST), Gangneung, 25451 Korea; 4 Division of Bio-Medical Science & Technology, University of Science and Technology (UST), Daejeon, 34113 Korea; 5 Department of Pharmacology, College of Medicine, The Catholic University of Korea, Seoul, 06591 Korea; 6 Department of Pharmacology, Yonsei University College of Medicine, Seoul, 03722 Korea; 7 Department of Internal medicine, Institute of Gastroenterology, Yonsei University College of Medicine, Seoul, 120-752 Korea; 8 Department of Surgery, Yonsei University College of Medicine, Seoul, 120-752 Korea; 9 Department of Surgery and Cancer, Imperial College London, London, W120NN UK; 10 Department of Biochemistry, College of Life Science and Biotechnology, Yonsei University, Seoul, 120-752 Korea

**Keywords:** Colorectal cancer, Epithelial-mesenchymal transition

## Abstract

WNT signaling activation in colorectal cancers (CRCs) occurs through *APC* inactivation or β-catenin mutations. Both processes promote β-catenin nuclear accumulation, which up-regulates epithelial-to-mesenchymal transition (EMT). We investigated β-catenin localization, transcriptome, and phenotypic differences of HCT116 cells containing a wild-type (HCT116-WT) or mutant β-catenin allele (HCT116-MT), or parental cells with both WT and mutant alleles (HCT116-P). We then analyzed β-catenin expression and associated phenotypes in CRC tissues. Wild-type β-catenin showed membranous localization, whereas mutant showed nuclear localization; both nuclear and non-nuclear localization were observed in HCT116-P. Microarray analysis revealed down-regulation of Claudin-7 and E-cadherin in HCT116-MT vs. HCT116-WT. Claudin-7 was also down-regulated in HCT116-P vs. HCT116-WT without E-cadherin dysregulation. We found that ZEB1 is a critical EMT factor for mutant β-catenin-mediated loss of E-cadherin and Claudin-7 in HCT116-P and HCT116-MT cells. We also demonstrated that E-cadherin binds to both WT and mutant β-catenin, and loss of E-cadherin releases β-catenin from the cell membrane and leads to its degradation. Alteration of Claudin-7, as well as both Claudin-7 and E-cadherin respectively caused tight junction (TJ) impairment in HCT116-P, and dual loss of TJs and adherens junctions (AJs) in HCT116-MT. TJ loss increased cell motility, and subsequent AJ loss further up-regulated that. Immunohistochemistry analysis of 101 CRCs revealed high (14.9%), low (52.5%), and undetectable (32.6%) β-catenin nuclear expression, and high β-catenin nuclear expression was significantly correlated with overall survival of CRC patients (*P = *0.009). Our findings suggest that β-catenin activation induces EMT progression by modifying cell-cell junctions, and thereby contributes to CRC aggressiveness.

## Introduction

WNT signaling pathway alterations play key roles in colorectal cancer (CRC) tumorigenesis. Specifically, WNT pathway activation promotes transcriptional activation of target genes involved in various tumorigenic processes, such as proliferation, stemness, and cell motility^1^. Up to 80% of sporadic CRCs show inactivating mutations or allelic loss of adenomatous polyposis coli (*APC*), is a key WNT pathway regulator, resulting in persistent WNT pathway activation^2^. Additionally, approximately 5% of sporadic CRCs show activating mutations in *CTNNB1* (β-catenin-encoding gene), which are extremely rare in CRCs with *APC* mutations. Although β-catenin mutations are infrequent in sporadic CRCs, these have been reported in about 18% of hereditary non-polyposis colorectal cancers (HNPCCs)^3,4^. Most β-catenin mutations occur in exon 3, which encodes an E3-ligase-binding region that helps β-catenin escape degradation and, consequently, results in WNT pathway activation^5^. Although it is unclear how cytosolic accumulation of β-catenin induces its translocation into the nucleus, both *APC* and β-catenin mutations commonly lead to nuclear overexpression of β-catenin^6^. Notably, since β-catenin is the acting downstream effector of WNT pathway, an enhanced understanding of its targeting and function could provide direct insight into how WNT activation promotes CRC tumorigenesis.

Nuclear β-catenin acts as a coactivator of T-cell and lymphoid enhancer factors (TCF-LEF), and thereby stimulates expression of target genes related to various oncogenic pathways, particularly the epithelial-to-mesenchymal transition (EMT). EMT results from β-catenin activation, and is directly associated with invasion and metastasis of various cancers^7^. During EMT, loss of cell junction molecules leads to perturbation of cell-cell interactions; this is considered the most critical step for cancer cells to dissociate from the primary tumor, invade surrounding tissues, and metastasize to secondary sites^8^. In normal cells, β-catenin promotes adherens junction (AJ) formation by binding to E-cadherin, but it can also function to induce EMT when released from the E-cadherin-β-catenin complex^9^. Notably, although the clinical significance of abnormal E-cadherin expression in prognosis, invasive potential, and metastasis of CRC is known, the expressional and functional relationship between E-cadherin and β-catenin remains poorly understood^10^.

In addition to AJs, tight junctions (TJs) play central roles in EMT regulation and subsequent cancer progression. In normal cells, TJs maintain cell polarity and integrity, but they are dismantled in cancers to allow dissemination. TJ proteins consist of three major groups: Claudins, Occludins, and linker molecules. Claudin and Occludin families facilitate tight sealing of cells in the epithelial sheet, whereas zonula occludins (ZO) protein-1, a linker molecule, mediates interaction between Claudins and Occludins and the actin cytoskeleton^11^. Of these TJ molecules, abnormal expression of several Claudin proteins (e.g., Claudin-1, -3, -4, and -7) has been associated with tumorigenesis of various cancers, including CRCs. Claudin expression has also been correlated with prognosis, invasion, and metastasis in CRCs. However, Claudin family members show heterogeneous expression patterns and even opposite roles in various types of cancers, and their expressional and functional relationships with β-catenin expression remain unclear^12^.

Here, we aimed to investigate the mechanism by which β-catenin activation affects cell-cell junctions during EMT progression using a panel of HCT116 cell lines with differential β-catenin mutation status.

## Materials and Methods

### Cell culture and reagents

HCT116 cell lines were purchased from Horizon Discovery (Cambridge, United Kingdom). HCT116 parental (HCT116-P) line contains one WT β-catenin allele and one mutant allele; HCT116-MT and HCT116-WT contain one mutant or one WT allele, respectively, generated by disruption of the other allele in the parent strain^13^. DLD-1, LoVo, RKO, HCT8, Hep3B, HepG2, and LS174T cell lines were purchased from the Korean Cell Line Bank (Cancer Research Institute, Seoul, Korea). DLD-1, LoVo, RKO, HCT8, LS174T, and HCT116 cells were maintained in Roswell Park Memorial Institute (RPMI) medium, containing 10% fetal bovine serum (FBS) and 1% penicillin/streptomycin (P/S) at 37 °C in a 5% CO_2_ incubator. Hep3B and HepG2 cells were respectively maintained in Dulbecco’s Modified Eagle Medium (DMEM) and Minimum Essential Medium (MEM), both containing 10% FBS and 1% P/S. For morphological analysis, images were obtained using the 20× objective of an Olympus (Tokyo, Japan) IX71 camera.

### Western blot analysis

Whole-cell lysates were prepared in Passive Lysis Buffer (Promega, Madison, WI, USA), containing protease inhibitor cocktail. Proteins were electrophoresed and transferred to a membrane. Membranes were incubated overnight at 4 °C with primary antibodies against GAPDH (Trevigen, Gaithersburg, MD, USA), β-catenin, E-cadherin (both from BD Biosciences, San Jose, CA, USA), FLAG (Sigma, St. Louis, MO, USA), EGFP, hnRNPC, α-tubulin (all from Santa Cruz Biotechnology, Santa Cruz, CA, USA), Claudin-7 (Thermo Fisher Scientific, Waltham, MA, USA), SNAIL, SLUG, TWIST1, ZEB1, TCF4, Vimentin, and N-cadherin (all from Cell Signaling Technology, Beverly, MA, USA). Membranes were then washed and incubated with appropriate secondary antibodies for 1 h at room temperature. Western blot images were analyzed using a LAS 4000 mini camera (Fujifilm, Tokyo, Japan), and relative band densities were quantified using ImageJ (NIH) software.

### Immunofluorescence

Cells grown on slides were rinsed with phosphate-buffered saline (PBS), fixed with 4% paraformaldehyde (PFA) or 100% methanol for 15 min, and permeabilized in 0.2% Triton X-100 in PBS. Fixed slides were incubated at 4 °C overnight with primary antibodies against β-catenin, E-cadherin (both from BD Biosciences), Claudin-7 (Thermo Fisher Scientific), or FLAG (Sigma). Appropriate fluorescence-labeled secondary antibodies were then applied for 1 h at room temperature (Invitrogen Life Technologies, Carlsbad, CA, USA). All images were collected using a LSM700 confocal microscope (Carl Zeiss, Oberkochen, Germany).

### Quantitative reverse transcription (qRT)-PCR

Total RNA was extracted using TRIzol Reagent (Life Technologies) according to manufacturer’s instructions. Reverse transcription was performed with 2-μg total RNA. Real-time PCR was performed using the ABI PRISM 7500 Sequence Detector (Applied Biosystems) and SYBR Premix Ex Taq II (TaKaRa, Kusatsu, Shiga Prefecture, Japan), according to manufacturer’s guidelines. All mRNA expression levels are presented as the mean ± standard deviation (SD) from two or three independent experiments. Melting curves of PCR products were assessed for quality control.

### Gene expression analysis

Microarray experiments were conducted on HumanHT-12 v4 Sentrix Expression BeadChip (Illumina, San Diego, CA, USA). Hybridization of labeled-cRNA to BeadChip, washing, and scanning were performed according to Illumina Bead Station 500X manual. Extraction of mRNA expression data and statistical analysis of raw data were performed using software provided by the manufacturer (Illumina GenomeStudio v2011.1). Expression intensities were normalized by quantile normalization technique; and using normalized intensities, genes differentially expressed in HCT116-WT and HCT116-MT cells were determined by the integrated statistical method previously reported^14^. Our microarray data are deposited in Gene Expression Omnibus (GSE126845). Differentially expressed genes (DEGs) were selected as those with *P* < 0.05 and fold change > 1.5. Functional enrichment analysis was performed using BINGO 2.3 plugin for Cytoscape software (http://www.psb.ugent.be/cbd/papers/BiNGO/Home.html), DAVID software (Database for Annotation, Visualization an Integrated Discovery, v6.7; http://david.abcc.ncifcrf.gov) to identify gene ontology (GO) biological processes, and Kyoto Encyclopedia of Genes and Genomes (KEGG) pathways represented by DEGs with statistical significance.

### Cell cycle analysis

HCT116-P, HCT116-WT, and HCT116-MT cells were washed with PBS and fixed in 95% ethanol. Cells were then incubated at 4 °C for 24 h, washed again, and stained in a solution of PBS, propidium iodide, and RNase A in the dark at 37 °C for 20 min. All samples were analyzed by flow cytometry using FACS Calibur (BD Biosciences) system.

### Luciferase assays

Wnt/β-Catenin signaling activity was measured using TOP/FOP-Flash luciferase activity assay. TOP-Flash contained a wild type LEF/TCF-binding site, while FOP-Flash contained a mutant LEF/TCF-binding site. HCT116 cells were transfected with reporter constructs (TOP-Flash or FOP-Flash). After 2 days, luciferase activity was measured using Dual-Luciferase Reporter Assay System Kit (Promega), according to manufacturer’s instructions. Results are presented as relative luciferase activity.

### Construction of β-catenin expression vectors and siRNAs for gene silencing

Coding sequences of β-catenin were cloned into a pLECE3 vector conjugated with EGFP at C-terminus. For generation of a β-catenin Ser45del mutant construct, substitution mutagenesis was performed at 45^th^ amino acid using β-catenin wild-type (WT) construct. An EGFP-conjugated E-cadherin expression vector was purchased from Addgene (#Plasmid 20089, Watertown, MA, USA). For gene silencing experiments, cells were transfected with short interfering RNAs (siRNAs) targeting *SNAI1*, *SNAI2*, *ZEB1*, and *TWIST1* (Bioneer, Daejeon, Korea). The siRNAs sequences are shown in Supplementary Table S1. Short hairpin RNAs (shRNAs) used in this study were obtained from a shRNA library of RNAi Consortium (TRC) provided by Yonsei genome center (Seoul, Korea).

### Tissue microarray construction

Representative areas were selected based on microscopic examination of hematoxylin and eosin (H&E)-stained slides, and corresponding spots were marked on the surface of the paraffin block. Using a manual tissue microarrayer, selected areas were removed, and 3-mm tissue cores were placed into 6 × 5 recipient blocks. Tissues from invasive tumors were then extracted; two tissue cores were processed from each sample to minimize extraction bias and ensure each case was represented in the final tissue microarray (TMA) block. Each core was assigned a unique TMA location number that is linked to a database with other clinicopathological data. After microtome sectioning and H&E staining, final TMA blocks contained cores from 101/101 (100%) specimens.

### Immunohistochemistry

Paraffin-embedded tissue blocks were cut into 4-µm sections. Immunohistochemical analysis was performed using a Ventana XT automated stainer (Ventana, Tucson, AZ, USA), with antibodies against β-catenin (1:200, BD biosciences), E-cadherin (1:1600, Abcam), and Claudin-7 (1:200, Sigma). The percentage of nuclear β-catenin staining was evaluated and categorized as negative, weak, or high, based on percentage of tumor cells with nuclear expression (0%, 1–29%, or ≥30%, respectively). The H-score method, which assigns an immunohistochemistry (IHC) H-score to each case on a continuous scale of 0–300 based on percentage of cells at different staining intensities, was used for the interpretation of Claudin-7 and E-cadherin results. Membranous staining was scored as follows: 0 for “no staining,” 1+ for “weak staining visible at high magnification,” 2+ for “intermediate staining,” and 3+ for “strong staining”. The percentage of cells at different staining intensities was determined by visual assessment, with scores calculated using the following formula: 1(% of 1+ cells) + 2(% of 2+ cells) + 3(% of 3+ cells)^15^. Samples were then classified as having low (H-score ≤ 200) or high (H-score > 200) protein expression.

### Wound-healing and invasion assays

For wound-healing assays, 0.5 × 10^6^ cells were seeded in 60-mm dishes and cultured until confluent. Using a (yellow) pipette tip, a straight scratch (wound) was generated, keeping the pipette tip at an angle of ~30°. Wound closure was then monitored over 48 h. For invasion assays, QCM 24-Well Cell Invasion assay kit was used (Millipore, Burlington, MA, USA), according to manufacturer’s guidelines. Briefly, insert interiors were rehydrated with pre-warmed serum-free media, and 0.3 × 10^6^ cells were added to each insert. Complete media (10% FBS) was added to the lower chambers, and cells were incubated for 48 h in a CO_2_ incubator. Cells were stained with 0.1% crystal violet solution, and images were obtained using the 4× or 10× objective.

### Tissue samples

A total of 101 stage III (metastasis to regional lymph nodes but not distant sites) CRC tissue samples that were treated with FOLFOX regimen were used in this study. This same cohort was analyzed in our previous study for molecular classification of CRCs^16^. All patients had undergone curative colorectal resection between 2006 and 2012, and none received neo-adjuvant chemotherapy. Specimens were obtained from the archives of Department of Pathology at Yonsei University in Seoul, Korea and from the Liver Cancer Specimen Bank of the National Research Resource Bank Program of the Korean Science and Engineering Foundation of the Ministry of Science and Technology. Patient data were collected retrospectively. All experimental protocols using patient tissues were approved by the Institutional Review Board of Yonsei University College of Medicine (approval number: 4-2018-0276) and conducted in accordance with all relevant policies, including obtaining informed consent from all subjects.

### Statistical analysis

Statistical analyses were performed using SPSS software, version 21.0.0.0 for Windows (IBM, Armonk, NY, USA). Mann-Whitney tests, Student’s t-tests, Fisher’s exact tests, or chi-square tests were used, depending on the purpose. Data are expressed as mean ± SD, and one-way analysis of variance (ANOVA) with a post hoc test (Bonferroni) was performed to compare multiple means. *P*-values < 0.05 were considered statistically significant.

### Ethical approval and informed consent

Authorization for the use of tissues for research purposes was obtained from the Institutional Review Board of Yonsei University College of Medicine (approval number: 4-2018-0276).

## Results

### HCT-116 cells containing a β-catenin mutation show nuclear β-catenin localization and Wnt pathway activation

**Figure 1:**
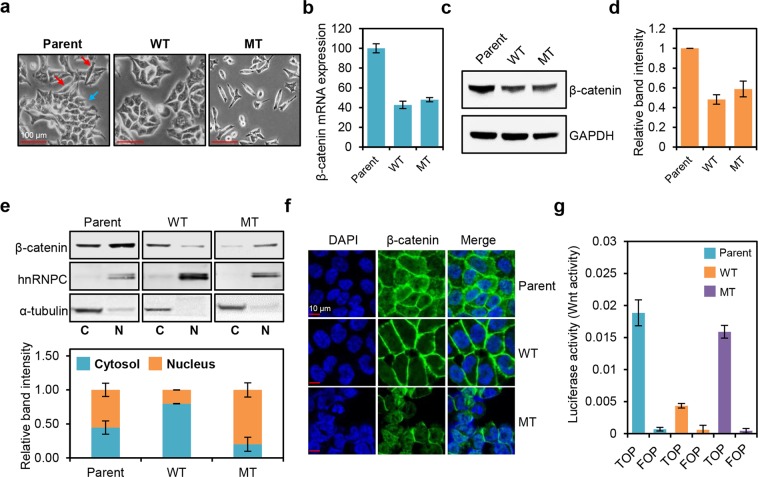
β-catenin mutation affects the localization and activation of β-catenin, as well as morphology of HCT116 cells. **(a)** Morphologies of HCT116 cells containing a wild-type (WT) β-catenin allele (HCT116-WT, WT), a mutant β-catenin allele (HCT116-MT, MT), or both wild-type and mutant alleles (HCT116-P, Parent). Red and blue arrows indicate mesenchymal-like and epithelial-like morphologies, respectively. **(b)** Relative β-catenin mRNA expression levels in HCT116 cell lines determined by qRT-PCR. **(c)** Western blot analysis of β-catenin protein levels in HCT116 cell lines. **(d)** Quantification of band intensities for the western blot shown in (**c**). **(e)** Top panel: western blot analysis of β-catenin levels in HCT116 cell lines subjected to cell fractionation. Bottom panel: quantification of band intensities for the western blot shown at top. hnRNPC and α-tubulin were used as nuclear and cytoplasmic markers, respectively. **(f)** Immunofluorescence microscopy analysis of β-catenin (stained in green) in HCT116 cells; DAPI staining is shown in blue. **(g)** Activation of WNT/β-catenin pathway in HCT116 cells, measured by TOP/FOP luciferase reporter assay. Error bars represent the standard deviation (SD) of the mean of two independent experiments. All assays shown in (**c**–**f**) were carried out in triplicate.

We utilized three HCT116 cell lines with differential β-catenin mutation status: parental HCT116 (HCT116-P) cells harboring one WT and one mutant allele (Ser45 del), HCT116-WT cells harboring one WT allele, and HCT116-MT cells harboring one mutant allele. As these lines are isogenic and differ only in their β-catenin mutation status, we considered this a proper model for investigating β-catenin mutation-induced phenotypes. Morphological analysis revealed clear differences. Specifically, HCT116-WT cells predominantly show epithelial-like morphology (round and organized sheet pattern), whereas HCT116-MT cells mostly display mesenchymal-like morphology (spindle-shaped and scattered pattern) (Fig. 1a). Both morphologies were heterogeneously observed in HCT116-P cells. Western blot and qRT-PCR analysis revealed similar levels of β-catenin mRNA and protein expression, respectively, in HCT116-WT and HCT116-MT cells, and these were about half the levels measured in HCT116-P cells, likely due to the single β-catenin allele in WT and MT lines (Fig. 1b–d).

To determine β-catenin localization in each line, we first performed cell fractionation and western blot analyses, which revealed that HCT116-WT cells mostly show non-nuclear β-catenin expression, whereas HCT116-MT cells specifically display nuclear β-catenin expression. HCT116-P cells showed both nuclear and non-nuclear expressions (Fig. 1e). Subsequent immunofluorescence microscopy analysis confirmed that WT β-catenin in HCT116-WT cells mostly localizes on the plasma membrane, whereas mutant β-catenin in HCT116-MT cells is specifically found in the nucleus. HCT116-P cells display a mixed pattern of membranous, cytoplasmic, and nuclear localization (Fig. 1f). Wnt pathway activation was also strongly up-regulated in both HCT116-P and HCT116-MT cells, as compared to HCT116-WT cells (Fig. 1g). We further analyzed the expression of Wnt/β-catenin signaling target genes, *CD44*, *MMP*7, *CCND1*, *CCNE2*, *BMP4*, and *AXIN2*. qPCR analysis showed that the expression of *BMP4*, *AXIN2*, and *CCNE2* was strongly up-regulated in HCT116-P and HCT116-MT cells compared to that of HCT116-WT cells. *CCND1* expression was slightly up-regulated in HCT116-MT and HCT116-P cells. On the other hand, *CD44* expression was higher in HCT116-WT than that in other cell lines, and *MMP7* expression was specifically up-regulated in HCT116-P cells (Supplementary Fig. S1). Overall, these results indicate that WT β-catenin is stably expressed on the cell surface where it participates in cell-cell junctions, whereas mutant β-catenin shows nuclear expression and functions as a transcriptional regulator of some of the reported Wnt/β-catenin signaling target genes according to the β-catenin mutation status in HCT116 cells.

### Cells expressing mutant β-catenin show enhanced expression of cell cycle-related genes and decreased expression of cell-cell adhesion pathway-related genes

**Figure 2:**
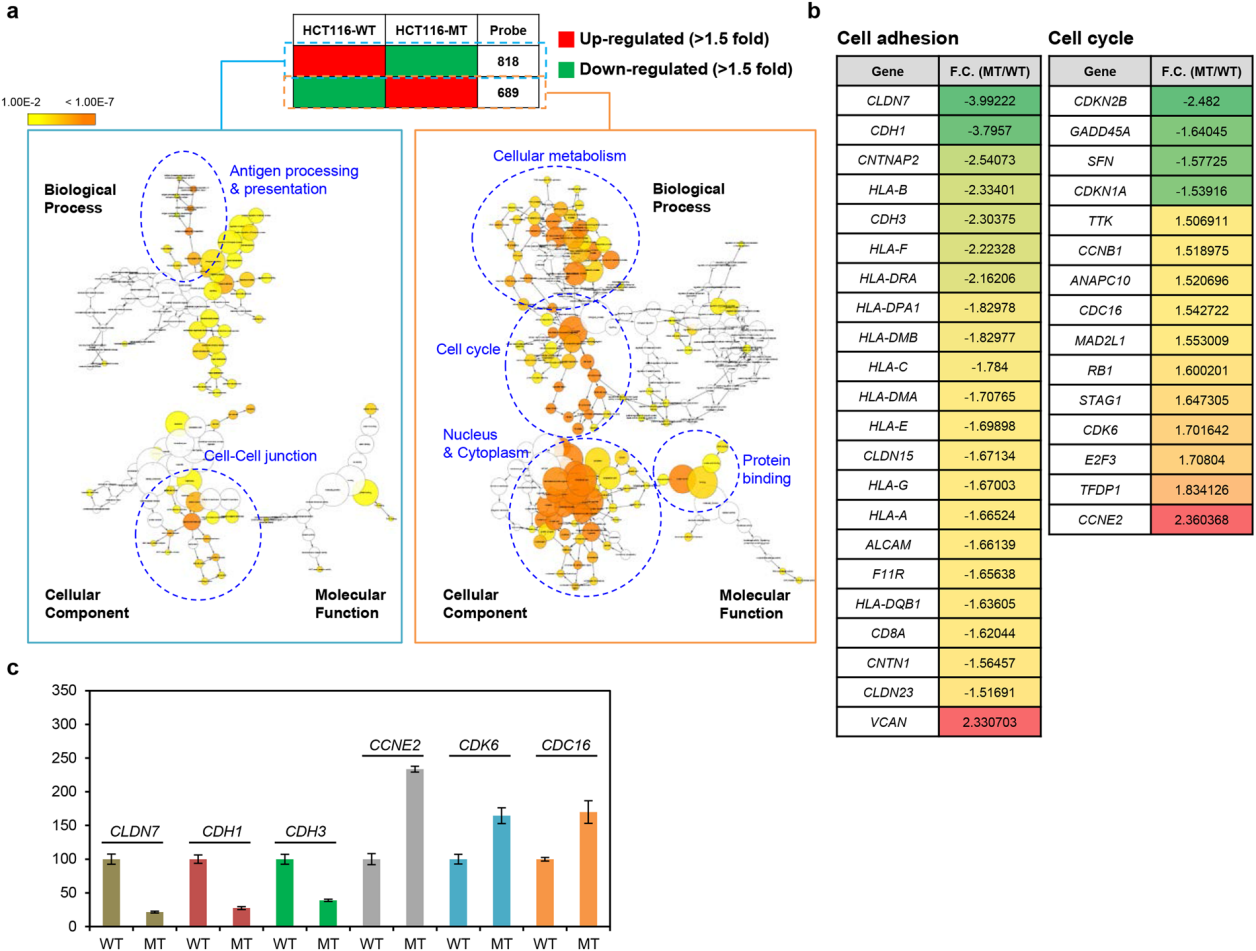
Cell cycle and cell-cell adhesion-related pathways were up- and down-regulated, respectively, in HCT116-MT cells, compared to HCT116-WT cells. **(a)** Transcriptional profiles of HCT116-WT and HCT116-MT cells were measured by Illumina HumanHT-12 v4 Expression BeadChip microarray. Gene ontology (GO) analysis was then performed on the list of 1,507 differentially expressed genes (DEGs) showing >1.5-fold up- or down-regulation in HCT116-MT vs. HCT116-WT cells with *P* < 0.05, using Cytoscape method to identify pathways enriched or suppressed in β-catenin mutant cells. **(b)** A curated gene list was obtained by overlapping the list DEGs identified by microarray analysis with a set of 128 cell cycle-related genes and a set of 134 cell adhesion-related genes obtained from the Kyoto Encyclopedia of Genes and Genomes (KEGG). Fold change (F.C.) in HCT116-MT relative to HCT116-WT cells is shown. **(c)** qRT-PCR validation of the microarray results was performed using six randomly selected genes. Error bars represent the SD of the mean of two independent experiments.

Since HCT116-WT and HCT116-MT cells show distinct patterns of β-catenin localization and cellular morphology, we performed microarray analysis on these lines to identify gene expression signatures that are dependent on β-catenin activation status and independent of other factors. We detected 1,507 DEGs (689 up-regulated, 818 down-regulated) displaying |fold change| >1.5 and *P*-value < 0.05 in HCT116-MT, as compared to HCT116-WT cells. Cytoscape GO analysis revealed HCT116-MT cells display significant up-regulation of cell cycle-related genes and significant down-regulation of cell-cell adhesion-related genes. Other cancer-related alterations (e.g., in cellular metabolism and antigen presentation genes) were also identified (Fig. 2a). Additional analysis using the ontology tool, DAVID, confirmed the up- and down-regulation, respectively, of cell cycle and cell-cell adhesion-related pathways in HCT116-MT cells (Supplementary Table S2). To narrow the list of candidate genes affected by β-catenin activation, we compared our 1,507 DEGs with known sets of 128 cell cycle-related genes and 134 cell adhesion-related genes from KEGG database. This enabled identification of 15 cell cycle-related and 22 cell adhesion-related candidate genes (Fig. 2b). Of these 37 genes, six were randomly chosen to validate our microarray data by qRT-PCR analysis, revealing consistent patterns of mRNA expression for each gene in both assays (Fig. 2c).

To confirm β-catenin activation promotes cell cycle up-regulation, we performed cell proliferation assays and flow cytometric analyses. As expected, HCT116-MT cells proliferated more rapidly than HCT116-WT cells, and HCT116-P cells showed an intermediate proliferation rate phenotype (Supplementary Fig. S2a). We further detected a 16.9% decrease and 16.8% increase in G1 and G2/M phases, respectively, for HCT116-MT vs. HCT116-WT cells; and a 9.8% decrease and 7.7% increase in G1 and G2/M phases, respectively, for HCT116-P vs. HCT116-WT cells. These findings indicate that HCT116-MT and HCT116-P cells show strong and intermediate cell cycle activation, respectively, as compared to HCT116-WT cells (Supplementary Fig. S2b).

Although EMT signature was not statistically significant in the pathway analysis results of our microarray data, we further analyzed expression of four main EMT factors, *SNAI1* (SNAIL-encoding gene), *SNAI2* (SLUG-encoding gene), *ZEB1*, and *TWIST1* by normalizing them to *GAPDH* expression. All of the four EMT factors were found to be up-regulated in HCT116-MT cells compared to HCT116-WT cells (*SNAI1*, 1.22 folds; *SNAI2*, 2.11 folds; *ZEB1*, 2.10 folds; *TWIST1*, 1.26 folds) (Supplementary Fig. S3a). We also compared our 1,507 DEGs with a set of 200 EMT-related genes obtained from KEGG database, which allowed us to obtain a list of 28 EMT-related genes, including strong up-regulation of *VIM* (vimentin) expression (7.57-fold increase) (Supplementary Fig. S3b). These findings suggest that expression of some EMT-related genes is significantly altered by β-catenin mutation.

### HCT116-MT cells show dual loss of tight and adherens junctions, whereas HCT116-P cells display only tight junction impairment

**Figure 3:**
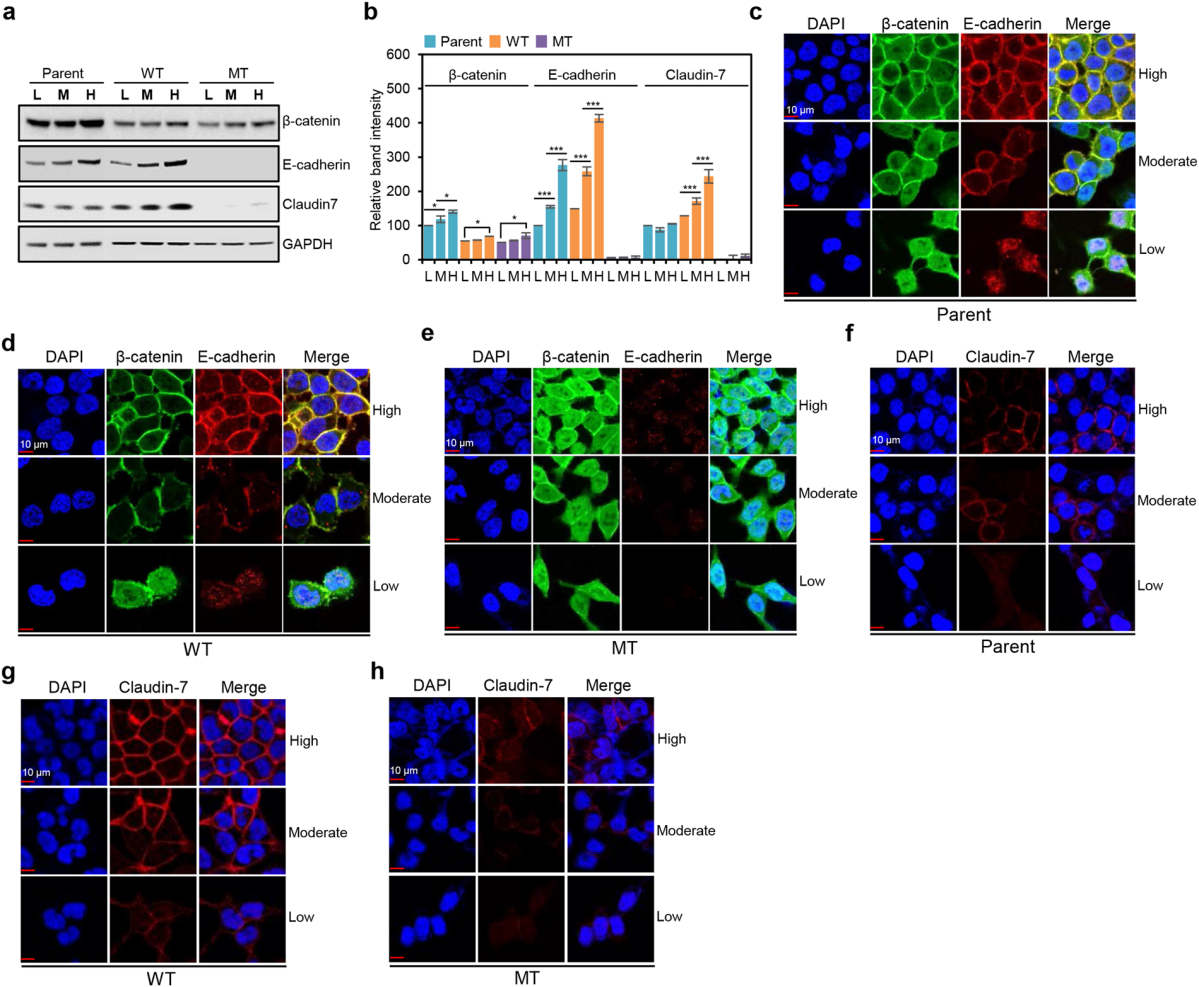
HCT116-P and HCT116-MT cells show dysregulation of cell-cell adhesion-related proteins. **(a)** Western blot analysis of β-catenin, Claudin-7, and E-cadherin levels in three HCT116 cell lines (Parent, WT, and MT). **(b)** Quantification of band intensities for the western blot shown in. (**a**) Error bars represent the SD of mean band intensities obtained from three independent experiments. One-way ANOVA with a post-hoc test (Bonferroni) was performed to compare multiple means; *P < 0.01, **P < 0.001, ***P < 0.0001. Statistical significance between low (L) and moderate (M) cell densities, and M and high (H) cell densities was shown. Statistical significance between L and H was shown when there was no statistical significance between L and M or M and H. Immunofluorescence microscopy analysis of β-catenin (stained in green) and E-cadherin (stained in red) in **(c)** HCT116-P, **(d)** HCT116-WT, and **(e)** HCT116-MT cells, under conditions of low, moderate, and high cell density. Immunofluorescence microscopy analysis of Claudin-7 (stained in red) in **(f)** HCT116-P, **(g)** HCT116-WT, and **(h)** HCT116-MT, under conditions of low, moderate, and high cell density. Nuclear DAPI staining is shown in blue. All assays were carried out in triplicate.

We next investigated β-catenin mutation-mediated alterations in cell-cell adhesion genes and found that, of the 22 adhesion-related genes identified by GO analysis, *CLDN7* and *CDH1* were most strongly down-regulated by β-catenin activation. *CLDN7* encodes Claudin-7 and *CDH1* encodes E-cadherin, which are well-known structural mediators that form TJ and AJ, respectively. We therefore evaluated their expression and localization in our three cell lines, under the conditions of low, moderate, and high cell density, as cell-cell junction molecules are known to show density-dependent expression^17^. Western blot analysis revealed a slight increase in β-catenin expression with increasing cell density in all HCT116 cell lines. For E-cadherin, HCT116-P and HCT116-WT cells show a pattern of gradually increasing cell density-dependent expression, whereas it is barely detectable in HCT116-MT cells. Claudin-7 is also highly expressed and increases with cell density in HCT116-WT cells; however, expression is much lower and unaffected by cell density in HCT116-P cells and is barely detectable in HCT116-MT cells (Fig. 3a,b). β-catenin, E-cadherin, and Claudin-*7* mRNA expression patterns mirror their protein expression patterns (Supplementary Fig. S4). These findings suggest impairment of Claudin-7-mediated TJ in HCT116-P cells and of both TJ and E-cadherin-dependent AJ in HCT116-MT cells.

To test cell-cell junction status in our cell lines, we performed immunofluorescence microscopy analysis. In HCT116-P and HCT116-WT cells at low density, E-cadherin and β-catenin showed poor colocalization and mostly nuclear β-catenin expression. At moderate cell density, β-catenin began to colocalize with E-cadherin on the cell surface, which was increasingly evident and organized at high density, indicating proper AJ formation (Fig. 3c,d). In HCT116-MT cells, however, E-cadherin was barely detectable at any cell density (Fig. 3e). For Claudin-7, HCT116-WT cells showed increasingly clear and organized membranous expression in a cell density-dependent manner, with gradual TJ formation. However, Claudin-7-mediated TJ appeared to be poorly formed in HCT116-P, irrespective of cell density, and protein expression was barely detected in HCT116-MT cells at any density (Fig. 3f–h).

To investigate the involvement of Wnt pathway in loss of cell-cell junction, we performed immunofluorescence microscopy analysis, western blot, and qPCR using HCT116-WT cells after Wnt3a treatment. Immunofluorescence microscopy and western blot analysis showed that 2-hour treatment of Wnt3a strongly induced concomitant translocation of β-catenin, SNAIL, ZEB1, and TWIST1 into the nucleus; and 4-hour treatment of Wnt3a led to downregulation of E-cadherin and Claudin-7 expressions (Supplementary Fig. S5a,b). Wnt3a treatment also induced transcriptional activation of downstream target genes of Wnt/β-catenin signaling, such as *CD44*, *AXIN2*, and *CCND1* (Supplementary Fig. S5c).

### E-cadherin binds to both WT and mutant β-catenin, and β-catenin is released from the cell membrane and degraded by loss of E-cadherin

**Figure 4:**
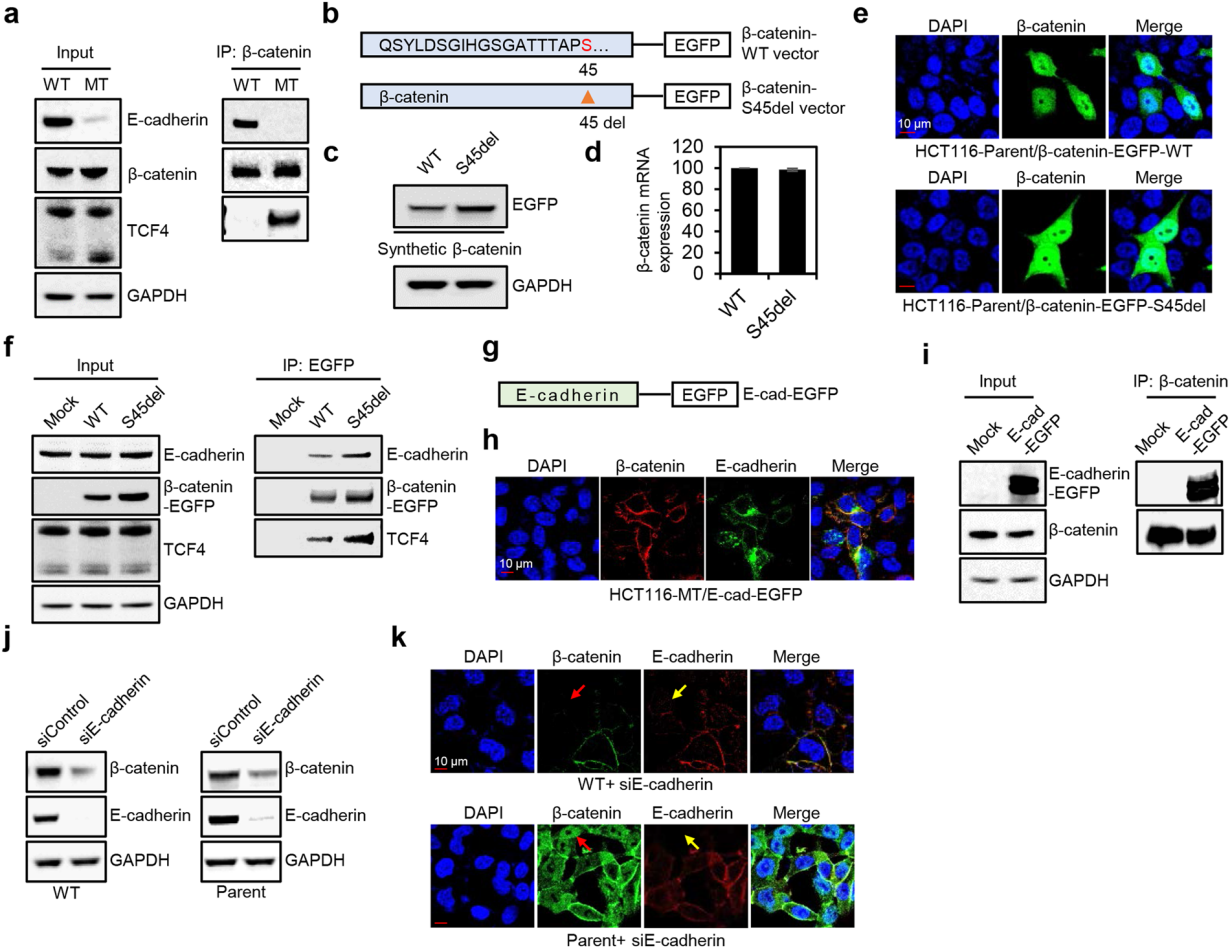
E-cadherin binded to both WT and mutant β-catenin, and knockdown of E-cadherin downregulated membranous β-catenin expression. **(a)** Immunoprecipitation was performed in HCT116-WT and HCT116-MT cells using a β-catenin antibody. **(b)** Schematic model of EGFP-conjugated β-catenin expression constructs. β-catenin-S45del vector was generated by mutagenesis using β-catenin-WT vector. **(c–e)** Western blot, qPCR, and immunofluorescence microscopy analysis of HCT116-P cells transfected with β-catenin-S45del vector or β-catenin-WT vector. **(f)** Immunoprecipitation was performed using an EGFP antibody in HCT116-P cells transfected with a control vector or β-catenin expression vectors. **(g)** Schematic model of EGFP-conjugated E-cadherin expression vector. **(h**,**i)** Immunofluorescence microscopy analysis and immunoprecipitation with a β-catenin antibody was performed in HCT116-MT cells transfected with E-cadherin expression vector. **(j**,**k)** Western blot and immunofluorescence microscopy analysis of HCT116-WT and HCT116-P cells transfected with siRNA against E-cadherin. Red and yellow arrows indicate loss of WT β-catenin and loss of E-cadherin, respectively. All assays were carried out in triplicate.

It is well-documented that E-cadherin sequesters β-catenin to the cell membrane, and thereby inhibits EMT progression^18,19^. Since there are no reports regarding the binding of E-cadherin with mutant β-catenin, we tested if E-cadherin binds to both WT and mutant β-catenin. Considering HCT116-MT cells exclusively show nuclear β-catenin expression, β-catenin binding to TCF4 was also tested. Immunoprecipitation was performed using a β-catenin antibody, and the binding of β-catenin with E-cadherin or TCF4 was analyzed in HCT116-WT and HCT116-MT cells. As expected, WT β-catenin was mainly bound to E-cadherin, while mutant β-catenin was mostly bound to TCF4 (Fig. 4a). To objectively compare binding affinity of WT and mutant β-catenin to E-cadherin, we generated EGFP-conjugated β-catenin expression constructs (a β-catenin-WT and β-catenin-S45del construct) (Fig. 4b). HCT116-P cells were transfected with β-catenin-WT or β-catenin-S45del vector. β-catenin-S45del vector showed stronger β-catenin expression compared to β-catenin-WT vector, due to resistance of mutant β-catenin to proteasomal degradation (Fig. 4c). mRNA expressions from both constructs were similar (Fig. 4d). Importantly, both synthetic WT and mutant β-catenin showed similar cellular localization (membranous, cytoplasmic, and nuclear) (Fig. 4e), and immunoprecipitation with an EGFP antibody showed that synthetic WT and mutant β-catenin bind to both TCF4 and E-cadherin, indicating that E-cadherin similarly binds to WT and mutant β-catenin (Fig. 4f). Next, we investigated if overexpression of E-cadherin in HCT116-MT cells could lead to the sequestration of mutant β-catenin to the cell membrane (Fig. 4g). Immunofluorescence microscopy analysis and immunoprecipitation with a β-catenin antibody showed that the induced expression of E-cadherin led to increased binding of mutant β-catenin to E-cadherin and its membranous localization (Fig. 4h,i). These findings suggest that binding loss of mutant β-catenin to E-cadherin might be caused by loss of E-cadherin expression. Since we also demonstrated that WT β-catenin at cell-cell junctions is as stably expressed as mutant β-catenin, we further tested if downregulation of E-cadherin expression using siRNA releases β-catenin from the cell membrane and leads to its degradation in HCT116-WT and HCT116-P cells. Immunofluorescence microscopy and western blot analysis showed that knockdown of E-cadherin downregulates the expression and membranous localization of β-catenin (Fig. 4j,k).

### HCT116 cells show differential migratory and invasive activity according to their β-catenin mutation status

**Figure 5:**
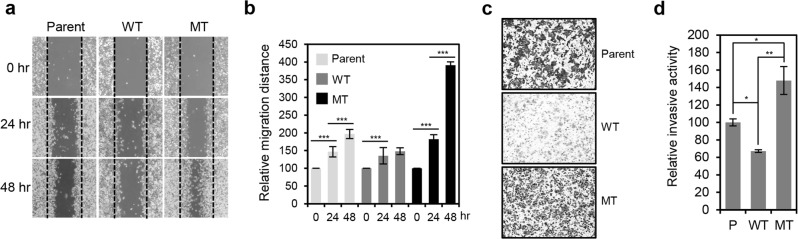
HCT116 cells showed differential migratory and invasive activity according to β-catenin mutations status. **(a)** Wound-healing assay performed using three HCT116 cell lines (Parent, WT, and MT). Gap closure was measured 24 and 48 h after initial scratch. **(b)** Quantification of relative migration distances for the wound-healing assay shown in. (**a**) Statistical significance between 0 and 24 hours, and 24 and 48 hours is shown. **(c)** Invasion assay performed using three HCT116 cell lines. Invasive activity was measured after 48 h by crystal violet staining. **(d)** Quantification of the relative number of stained cells in (**c**) using ImageJ software. Statistical significance between HCT116-P, HCT116-WT, and HCT116-MT is shown. All assays were carried out in triplicate.

To evaluate migratory activity of HCT116 cells with differential β-catenin mutation status and dysregulation of cell-cell junctions, we performed wound-healing assays, measuring gap healing at 24 and 48 h after initial scratch. We found that wound-healing rate is significantly higher for HCT116-P than HCT116-WT cells, and the highest overall for HCT116-MT cells (Fig. 5a,b). We then performed invasion assays and found HCT116-MT and HCT116-P cells are highly invasive compared to HCT116-WT cells, with HCT116-MT cells showing the highest invasive activity (Fig. 5c,d). This suggests that β-catenin activation and loss of cell-cell junctions are directly associated with mesenchymal-like features of HCT116 cells.

### β-catenin/ZEB1 axis plays important roles in loss of cell-cell junction in both HCT116-P and HCT116-MT cells

**Figure 6:**
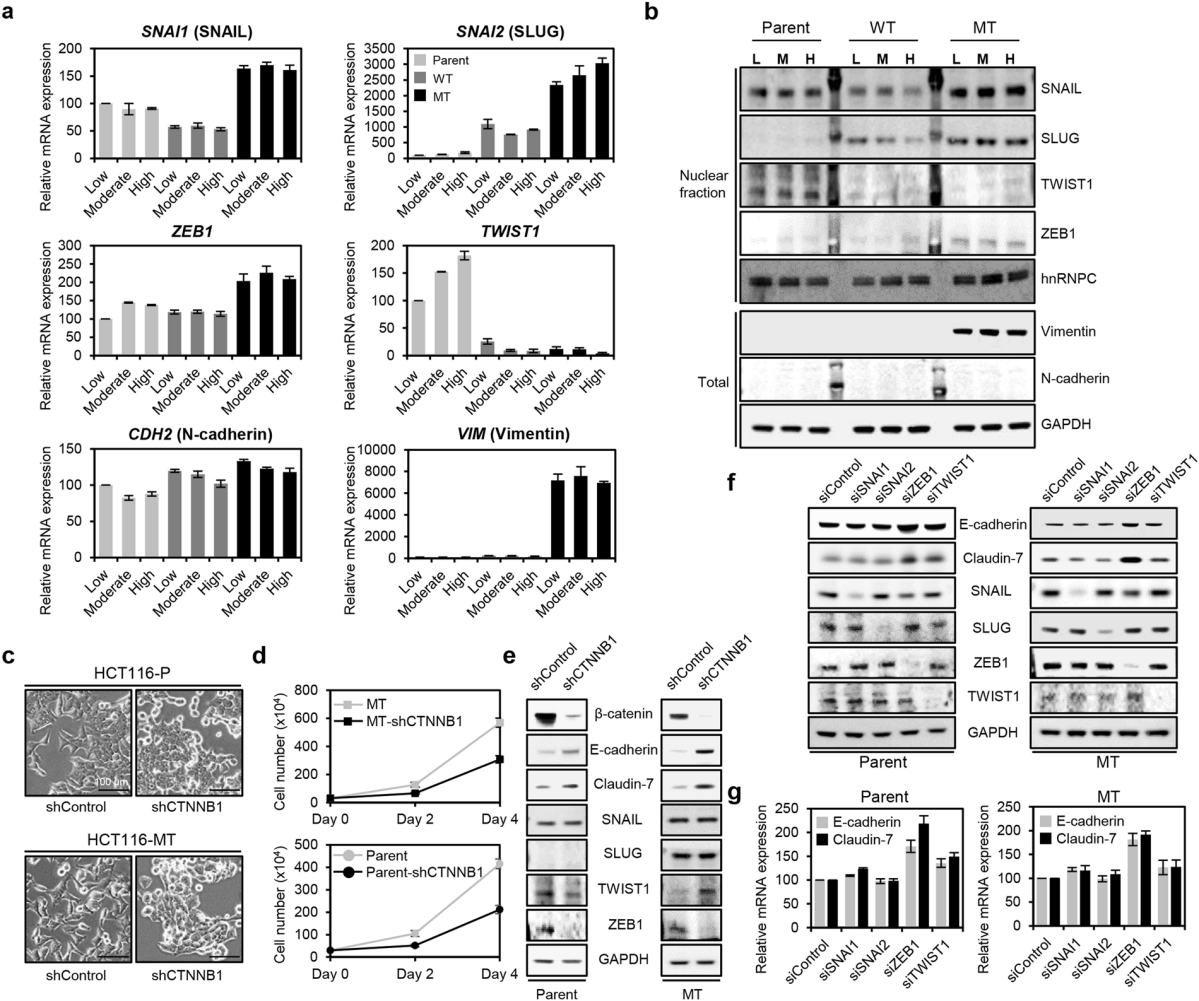
ZEB1 played important roles in mutant β-catenin-mediated loss of cell-cell junction molecules. **(a**,**b)** Cell density-dependent mRNA and protein expression of six EMT markers (*SNAI1*, *SNAI2*, *ZEB1*, *TWIST1*, *VIM*, and *CDH2*) measured by qRT-PCR and western blot in HCT116-P, HCT116-WT, and HCT116-MT cells. Error bars represent the SD of the mean of two independent experiments. **(c)** Morphologies of HCT116-P and HCT116-MT cells after complete knockdown of β-catenin expression using shRNA. **(d)** A proliferation assay was performed using HCT116-P and HCT116-MT cells with or without β-catenin expression. 0.3 × 10^6^ cells were seeded in 60-mm dishes and manually counted after 2 and 4 days. **(e)** Western blot analysis of E-cadherin, Claudin-7, SNAIL, SLUG, ZEB1, and TWIST1 in HCT116-P and HCT116-MT cells with or without β-catenin ablation. **(f**,**g)** Western blot and qPCR analysis of E-cadherin and Claudin-7 after knockdown of SNAIL, SLUG, ZEB1, and TWIST1 in HCT116-P and HCT116-MT cells. Error bars represent the SD of the mean of two independent experiments. All assays were carried out in duplicate.

Although both HCT116-P and HCT116-MT cells display mesenchymal-like behaviors, they show distinct cell morphology and motility patterns, which are mainly attributable to β-catenin mutation status (Figs. 1a and 5a–d). To more precisely elucidate EMT status in HCT116-P and HCT116-MT cells, we measured mRNA expression of *SNAI1*, *SNAI2*, *ZEB1*, *TWIST1*, *VIM*, and *CDH2* (N-cadherin coding gene) under conditions of increasing cell density. Using qRT-PCR, we detected *SNAI1* mRNA expression levels that are 2- and 3-fold higher, respectively, in HCT116-P and HCT116-MT cells than in HCT116-WT cells, at all densities (Fig. 6a). *SNAI2* mRNA was also up-regulated by about 2.5-fold in HCT116-MT compared to HCT116-WT cells, although the lowest expression was detected in HCT116-P cells (down 6-fold vs. HCT116-MT). Compared to HCT116-WT cells, *ZEB1* mRNA expression was slightly up-regulated in HCT116-P cells, whereas about 2-fold up-regulation was detected in HCT116-MT cells. In contrast, *TWIST1* expression was strongly up-regulated only in HCT116-P cells in a cell density-dependent manner. *CDH2* expression was slightly higher in HCT116-MT cells, compared to that of HCT116-P and HCT116-WT cells. Notably, *Vim* expression was selectively and strongly up-regulated in HCT116-MT cells. Similar protein expression patterns were also detected for each EMT marker (Fig. 6b). These data suggest that HCT116-MT cells show more progressed EMT than HCT116-P cells, in terms of both motility and EMT marker expression.

Next, we sought to investigate the relationship between mutant β-catenin, EMT factors, and loss of cell-cell junction. To identify EMT transcription factors directly affected by β-catenin, we ablated β-catenin expression in HCT116-P and HCT116-MT cells using lentiviral-encoded short hairpin RNA (shRNA). Notably, HCT116-P and HCT116-MT cells with repressed β-catenin expression commonly showed epithelial-like morphology (Fig. 6c), and the growth rate of HCT116-P and HCT116-MT cells with ablated β-catenin expression was much lower than their counterpart cells with normal β-catenin expression (Fig. 6d). Then, we measured the expression of EMT factors by western blot. Complete knockdown of β-catenin barely affected the expression of SNAIL and SLUG in both cell lines, whereas complete loss of ZEB1 with decrease of TWIST1 expression and complete loss of ZEB1 with increase of TWIST1 expression were observed in HCT116-P and HCT116-MT cells, respectively. Importantly, knockdown of β-catenin led to strong increase of E-cadherin and Claudin-7 expression (Fig. 6e). These findings suggest that ZEB1, a direct target gene of β-catenin, might be functionally important in terms of the mutant β-catenin mediated downregulation of cell-cell junction molecules in both HCT116-P and HCT116-MT cells. Next, we analyzed the expression of E-cadherin and Claudin-7 in HCT116-P and HCT116-MT cells after knockdown of SNAIL, SLUG, ZEB1, or TWIST1 by siRNA. Western blot and qPCR analysis showed that ZEB1 knockdown strongly increases the expression of E-cadherin and Claudin-7 in both cell lines, while TWIST1 knockdown slightly up-regulates the expression of E-cadherin and Claudin-7 only in HCT116-P cells. Downregulation of SNAIL or SLUG barely affected expression of E-cadherin and Claudin-7 (Fig. 6f,g).

### Claudin-7 down-regulation in HCT116 cells is critical for acquisition of mesenchymal-like features

**Figure 7:**
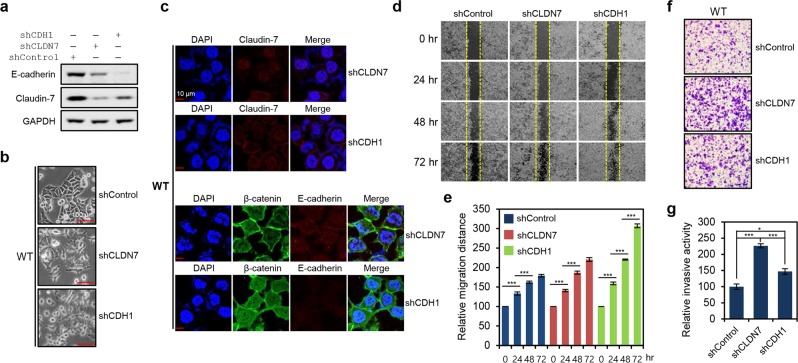
Loss of tight junctions mediated by Claudin-7 dysregulation was sufficient for acquisition of mesenchymal-like features by HCT116 cells. **(a)** Western blot analysis of E-cadherin and Claudin-7 expression in HCT116-WT cells with stable shRNA-mediated knockdown of Claudin-7 (shCLDN7) or E-cadherin (shCDH1). **(b)** Morphological changes in HCT116-WT cells after stable knockdown of Claudin-7 or E-cadherin. Cell images were obtained using the 20x objective. **(c)** Immunofluorescence microscopy analysis of Claudin-7 (stained in red), β-catenin (stained in green), and E-cadherin (stained in red) in HCT116-WT cells with stable shRNA-mediated knockdown of Claudin-7 or E-cadherin knockdown. **(d)** Wound-healing assay performed using HCT116-WT cells with stable knockdown of Claudin-7 or E-cadherin. Gap closure was measured at 24, 48, and 72 h after the initial scratch, due to the relatively low migratory activity of HCT116-WT. **(e)** Quantification of relative migration distances for the wound-healing assay shown in. (**d**) Statistical significance between 0 and 24 hours, 24 and 48 hours, and 48 and 72 hours is shown. **(f)** Invasion assay performed using HCT116-WT cells with stable knockdown of Claudin-7 or E-cadherin. Invasive activity was measured after 48 h by crystal violet staining. **(g)** Quantification of the relative number of stained knockdown cells in (**f**) using ImageJ software. Statistical significance between shControl, shCLDN7, and shCDH1 is shown. Error bars in (**e**,**g**) represent the SD of the mean of results from three independent experiments. All assays were carried out in triplicate.

In order to confirm whether mesenchymal-like features of HCT116-MT and HCT116-P cells directly result from β-catenin activation-mediated down-regulation of cell-cell junction molecules, we generated stable Claudin-7 or E-cadherin knockdown HCT116-WT cells using shRNA. Protein knockdown was confirmed by western blot, and we found that Claudin-7 knockdown promotes decreased E-cadherin expression and vice versa (Fig. 7a). Critically, both Claudin-7 and E-cadherin knockdown cells showed mesenchymal-like morphology, displaying multiple lamellipodia at their leading edges to facilitate migration (Fig. 7b). Immunofluorescence analysis further revealed disturbed cell-cell interactions and interdependent loss of Claudin-7 and E-cadherin expression in stable knockdowns (Fig. 7c). Wound-healing was significantly accelerated in both Claudin-7 and E-cadherin knockdown cells, showing more rapid closure in E-cadherin knockdowns (Fig. 7d,e). Enhanced invasion was also observed in both knockdowns, with a higher activity observed in Claudin-7 knockdown cells (Fig. 7f,g). These findings indicate interdependence between AJ and TJ formation, and suggest that dysregulation of TJs is sufficient to induce mesenchymal-like features in HCT116 cells.

### Loss of E-cadherin leads to dramatic up-regulation of migratory and invasive activity in HCT116-P cells

**Figure 8:**
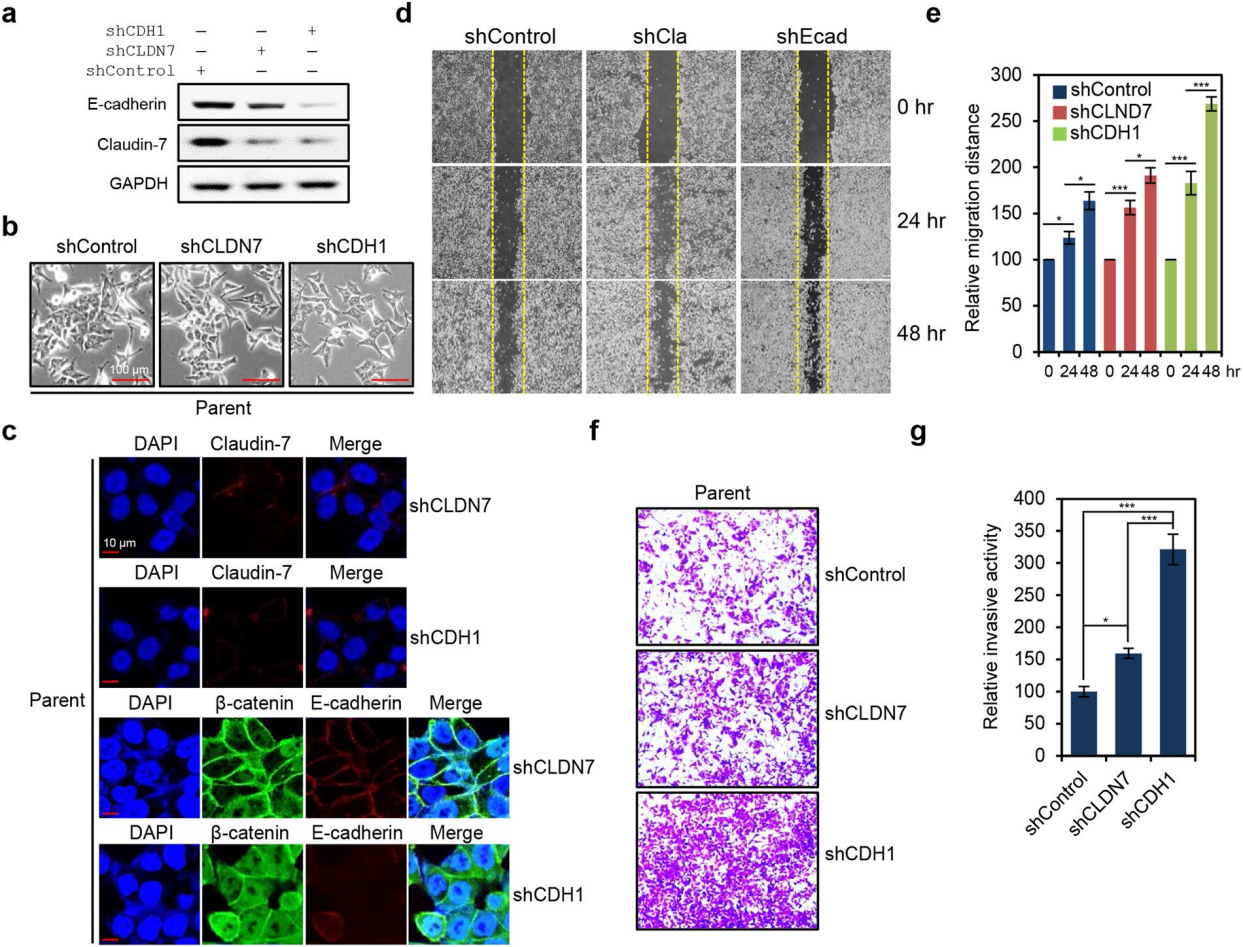
Additional loss of adherens junctions (AJs) led to EMT progression in HCT116-P cells. **(a)** Western blot analysis of Claudin-7 and E-cadherin expression in HCT116-P cells with stable shRNA-mediated knockdown of Claudin-7 (shCLDN7) or E-cadherin (shCDH1). **(b)** Morphological changes in HCT116-P cells after stable knockdown of Claudin-7 or E-cadherin. **(c)** Immunofluorescence microscopy analysis of Claudin-7 (stained in red), β-catenin (stained in green), and E-cadherin (stained in red) in HCT116-P cells with stable shRNA-mediated knockdown of Claudin-7 or E-cadherin knockdown. **(d)** Wound-healing assay performed on HCT116-P cells with stable knockdown of Claudin-7 or E-cadherin. **(e)** Quantification of relative migration distances for the wound-healing assay knockdown shown in. (**d**) Statistical significance between 0 and 24 hours, and 24 and 48 hours is shown. **(f)** Invasion assay performed on HCT116-P cells with stable knockdown of Claudin-7 or E-cadherin. **(g)** Quantification of the relative number of knockdown stained cells in (**f**) by ImageJ software. Error bars in (**e**,**g**) represent the SD of the mean of results from three independent experiments. Statistical significance between shControl, shCLDN7, and shCDH1 is shown. All assays were carried out in triplicate.

Our data indicate that Claudin-7 is down-regulated in HCT116-P compared to HCT116-WT cells and does not increase with cell density. Furthermore, levels of membranous E-cadherin in HCT116-P cells are similar to those observed in HCT116-WT cells. We therefore tested whether Claudin-7-mediated TJs are indeed impaired in HCT116-P cells, and if additional loss of E-cadherin further up-regulates migratory and invasive activity of these cells. To this end, we generated stable shRNA-knockdowns of Claudin-7 or E-cadherin in HCT116-P knockdown cells. Protein knockdown was confirmed by western blot. We further found that Claudin-7 knockdown promotes a slight decrease in E-cadherin expression and vice versa (Fig. 8a). Additionally, no clear morphological changes were observed in Claudin-7 knockdowns, whereas E-cadherin knockdown cells showed disturbed cell polarization and adjoining (Fig. 8b). These morphological and expressional changes were also observed during immunofluorescence microscopy analysis (Fig. 8c). We also found that E-cadherin knockdown strongly induces HCT116-P cell migration, whereas migration is only slightly enhanced by Claudin-7 knockdown (Fig. 8d,e). Consistent with this, invasive activity was strongly up-regulated in E-cadherin knockdowns, but was induced to a much lesser extent in Claudin-7 knockdown cells (Fig. 8f,g). These findings indicate that AJs are minimally affected by further loss of TJs in HCT116-P cells due to pre-existing TJ impairment, and loss of AJs leads to a more advanced EMT phenotype in those cells.

### Clinicopathologic and molecular features of CRCs with nuclear β-catenin expression

Our findings suggest that β-catenin mutations promote its nuclear expression, leading to Claudin-7 and E-cadherin down-regulation. We therefore examined the association between nuclear β-catenin expression and expression of Claudin-7/E-cadherin in 101 stage III CRC tissues by IHC. We exclusively detected membrane-associated β-catenin in crypt cells of normal colon mucosa, whereas CRCs showed heterogeneous nuclear accumulation of β-catenin that can be categorized as follows: 1) no nuclear expression, similar to normal tissues, 33/101 samples; 2) low nuclear expression (1–29% of tumor cells), 53/101 samples; and 3) high nuclear expression (≥30% of tumor cells), 15/101 samples (Supplementary Fig. S6a,b).

We then assessed clinicopathologic features of CRC tissues showing negative, low, and high nuclear β-catenin expressions (Supplementary Table S3), and found that those with high expression showed significantly larger tumor size (*P* = 0.028) and decreased prevalence of microsatellite instability-high (MSI-H) (*P* = 0.013). We could not find statistical significance between high nuclear β-catenin expression and expression of Claudin-7 (*P* = 0.474) and E-cadherin (*P* = 0.093), probably due to the small case number of CRCs with high nuclear β-catenin expression (Supplementary Fig. S6c and Supplementary Table S3). In the same context, no significant relationship was found between the mean percentage of nuclear β-catenin expression in CRCs showing metastasis or no metastasis after operation (*P* = 0.078) (Supplementary Fig. S6d). Notably, Kaplan–Meier survival analysis showed that overall survival (*P* = 0.009) of CRC patients with negative and low nuclear β-catenin expression is significantly longer than that of patients with high nuclear β-catenin expression (Supplementary Fig. S7).

## Discussion

As a key downstream WNT pathway effector, β-catenin plays known roles in proliferation, survival, and EMT of CRC cells. EMT is particularly important due to its involvement in loss of cell-cell adhesion and gain of migratory and invasive features, which render some cancers incurable by current therapeutic regimens^20^. Indeed, most cancer-related deaths are attributable to metastatic diseases. Loss of cell-cell junctions is critical for EMT progression; and although its clinical significance has been reported in CRC^21^, relationship between loss of cell-cell junction and β-catenin-mediated EMT is poorly understood. Therefore, elucidation of mechanisms underlying β-catenin-mediated EMT is critical for understanding metastasis in a subset of CRCs.

In most CRCs, β-catenin stabilization results from mutations in *APC*. However, as APC is involved in various cellular functions, independent of β-catenin^22^, it is difficult to assess specific consequences of β-catenin activation in *APC* mutant cells. Therefore, in this study, we sought to elucidate the mechanism of β-catenin activation-mediated EMT using isogenic HCT116 cell lines with differential β-catenin mutation status. To validate our strategy, we indeed evaluated the localization and activation status of β-catenin in two APC mutant CRC cell lines (DLD-1 and LoVo), two CRC cell lines with no Wnt pathway relevant mutations (RKO and HCT8), a hepatocellular carcinoma (HCC) cell line with an *AXIN1* mutation (Hep3B), a HCC cell line with a *CTNNB1* mutation (HepG2), and a CRC cell line with a *CTNNB1* mutation (LS174T). Immunofluorescence microscopy and western blot analysis showed that CRCs with *APC* mutations more clearly display nuclear β-catenin localization compared to CRC cells with no Wnt pathway relevant mutations and HCC cells with an *AXIN1* mutation. Importantly, HCC and CRC cells with a *CTNNB1* mutation commonly showed much stronger nuclear β-catenin localization, compared to CRC cells with *APC* mutations (Supplementary Fig. S8a,b). Wnt pathway activity was also found to be proportional to the level of nuclear β-catenin expression in each cell line (Supplementary Fig. S8c). These findings suggest that localization and activation status of β-catenin could be differentially affected by *APC* or *CTNNB1* mutations, and cell lines with *APC* mutation might not be a proper model for investigating β-catenin specific roles in cancer cells.

We found that WT and mutant β-catenin show distinct localization on the cell membrane and in the nucleus, respectively. Furthermore, despite the well-known model that WT β-catenin is continuously degraded in the absence of either WNT ligand or β-catenin mutations, HCT116-WT and HCT116-MT cells showed similar levels of β-catenin expression. We also demonstrated that E-cadherin similarly binds to WT and mutant β-catenin, and WT β-catenin is stable when bound to E-cadherin and subjected to degradation when released from E-cadherin. This suggests WT β-catenin at cell-cell junctions is as stably expressed as mutant β-catenin in the nucleus, and that protein function is likely to be dependent on localization and E-cadherin. Indeed, we found that WT β-catenin promotes E-cadherin-dependent AJ formation, whereas nuclear-localized mutant β-catenin induces partial and progressed EMT, with an accompanying dysregulation of TJ and AJ. Since strong AJs inhibit EMT progression, WT β-catenin thereby plays a vital role in maintaining epithelial-like behavior.

Notably, a mixed pattern of β-catenin expression was observed in HCT116-P cells, which showed intermediate mesenchymal-like features. In particular, the strong TJs and AJs observed in HCT116-WT cells were absent in HCT116-MT cells, whereas HCT116-P cells only showed impairment of TJs, which likely promoted their intermediate invasive and migratory activities. Recent studies have suggested EMT is not an all-or-nothing process, but rather arises from multi-step changes, displaying numerous intermediate phenotypes. These have been referred to by terms such as “intermediate EMT” and incomplete EMT”, and “partial EMT^23–27^”. Our results suggest that HCT116-P cells represent a partial EMT phenotype, with HCT116-MT cells showing more progressed EMT. Consistent with this, of the six tested EMT-inducing factors, HCT116-P cells showed a unique and specific pattern of *TWIST1* up-regulation with slight up-regulation of *SNAI1* and *ZEB1* expression, whereas HCT116-MT cells showed concomitant and strong up-regulation of *SNAI1*, *SNAI2*, *ZEB1*, and *VIM* expression with slight up-regulation of *CDH2*. We also demonstrated that ZEB1 and TWIST1 play key roles in down-regulation of E-cadherin and Claudn-7 expressions in HCT116-P cells, while ZEB1 exclusively does the same in HCT116-MT cells. In addition, it is well-documented that adherens junction formed by β-catenin/E-cadherin complex inhibits EMT progression^18,19^. Since HCT116-P cells express both WT and mutant β-catenin, they are undergoing E-cadherin/WT β-catenin complex-mediated EMT inhibition and nuclear mutant β-catenin-mediated EMT progression. We propose that the combined form of EMT inhibition and progression axes displays the incomplete or partial EMT.

Emerging roles for TJs, particularly those involving Claudin family members, have been uncovered in various cancers, including gastric cancers, breast cancers, and CRCs. TJ loss is required for cells to overcome the barriers imposed by TJ-bound epithelial tissues and metastasize to surrounding tissues^28^. Both up- and down-regulation of Claudin-7 expressions have been reported in CRCs, and are linked to invasion, metastasis, and disease prognosis^29–32^. However, these findings are inconsistent and limited to the analysis of Claudin-7 alone, without considering nuclear β-catenin expression. Here, we detected Claudin-7 down-regulation in HCT116-P and HCT116-MT cells harboring a mutant β-catenin allele. Due to the combined loss of AJ and TJ in HCT116-MT cells, we used HCT116-P cells as a model to evaluate EMT phenotypes induced by TJ loss. These displayed poorly formed TJ and were more invasive and migratory than HCT116-WT cells. We further demonstrated that shRNA-knockdown of Claudin-7 is sufficient to induce partial EMT in HCT116-WT cells. Claudin-7-knockdown HCT116-WT cells displayed clear morphological and motility features associated with EMT. Moreover, they expressed decreased E-cadherin levels, suggesting interdependence between TJ and AJ. As described above, HCT116-P cells represent a partial EMT state due to intact AJs. Indeed, E-cadherin knockdown further promotes invasive and migratory activity in HCT116-P cells, whereas knockdown of Claudin-7 has only minor effects. These findings indicate that β-catenin-mediated TJ modulation is sufficient to induce at least partial EMT, and additional dysregulation of AJ is required for further EMT progression.

Results of studies assessing the clinical significance of nuclear β-catenin expression in CRCs had numerous inconsistencies^6,33–35^. These could have resulted from the highly heterogeneous β-catenin expression patterns observed in CRCs, which we noted in our cohort of patient tissues. Based on the observation that ~50% nuclear β-catenin expression is sufficient to induce dysregulation of Claudin-7 but not E-cadherin in HCT116-P cells, we predicted that E-cadherin loss is likely to be detected only in CRC tissues with extremely high nuclear β-catenin expression. However, in our 101 CRC samples, we could not find a significant correlation between nuclear β-catenin expression and expression of Claudin-7 or E-cadherin. The discrepancies between our cellular experiments and human tissue data might have resulted from any of the following: 1) β-catenin-independent mechanisms for regulation of Claudin-7 and E-cadherin expression; 2) highly heterogeneous membranous and nuclear β-catenin expression in CRCs; or 3) small number of cases with high nuclear β-catenin expression. Therefore, we tested for inverse expressional correlations between β-catenin and Claudin-7/E-cadherin in solid-pseudopapillary neoplasm (SPN) tissues. These have homogenous mutations in exon 3 of β-catenin in nearly all tumor cells, leading to homogenous β-catenin nuclear expression^36^. Immunohistochemical analysis of 15 SPN samples revealed almost 100% nuclear β-catenin expression, accompanying complete loss of both Claudin-7 and E-cadherin (Supplementary Fig. S9). Additionally, although CRC tissues were not a perfect model for elucidating the relationship between β-catenin, Claudin-7, and E-cadherin due to heterogeneous β-catenin nuclear expression, we found a tendency toward frequent down-regulation of Claudin-7 and E-cadherin, and higher incidence of metastasis in CRCs with elevated β-catenin nuclear expression. Our findings suggest a model whereby β-catenin mutations and consequent nuclear expression lead to partial or complete loss of cell-cell junctions in an EMT factor-dependent manner, inducing EMT progression (Supplementary Fig. S10).

## Supplementary information

Supplementary Information

## Data Availability

All data analyzed in this study are presented in this article and also available upon request.
