# Supplementary material for: β-catenin activation down-regulates cell-cell junction-related genes and induces epithelial-to-mesenchymal transition in colorectal cancers
Source: Sci Rep. 2019 Dec 5;9:18440. doi: 10.1038/s41598-019-54890-9 (PMC6895046; doi:10.1038/s41598-019-54890-9)

β-catenin activation down-regulates cell-cell junction-related genes and induces epithelial-to-mesenchymal transition in colorectal cancers

Won Kyu Kim[1,2,3], Yujin Kwon[1,2,3,4], Mi Jang[2], Minhee Park[1,2], Jiyoon Kim[5], Suyeon Cho[3,4], Dong Geon Jang[1,6], Wook-Bin Lee[3], Sang Hoon Jung[3,4], Hye Jin Choi[7], Byung Soh Min[8], Tae Il Kim[7], Sung Pil Hong[9], Young-Ki Paik[10], Hoguen Kim[1,2,*]

Won Kyu Kim and Yujin Kwon equally contributed to this article.

[1]Brain Korea 21 PLUS Project for Medical Science

[2]Departments of Pathology, Yonsei University College of Medicine, Seoul 120-752, Korea

[3]Natural Product Research Center, Korea Institute of Science and Technology (KIST), Gangneung 25451, Korea

[4]Division of Bio-Medical Science & Technology, University of Science and Technology (UST), Daejeon 34113, Korea

[5]Department of Pharmacology, College of Medicine, The Catholic University of Korea, Seoul, 06591, Korea

[6]Department of Pharmacology, Yonsei University College of Medicine, Seoul 03722, Korea.

[7]Department of Internal medicine, Institute of Gastroenterology, Yonsei University College of Medicine, Seoul 120-752, Korea

[8]Department of Surgery, Yonsei University College of Medicine, Seoul 120-752, Korea

[9]Department of Surgery and Cancer, Imperial College London, London, W120NN, UK

[10]Department of Biochemistry, College of Life Science and Biotechnology, Yonsei University, Seoul 120-752, Korea

***Corresponding author:**

Hoguen Kim, M.D., Ph.D.

Professor of Pathology

Yonsei University College of Medicine, Seoul 120-752, Korea

Tel: +82 2 2228 1761, Fax: +82 2 363 5263

e-mail: hkyonsei@yuhs.ac

Supplementary Information

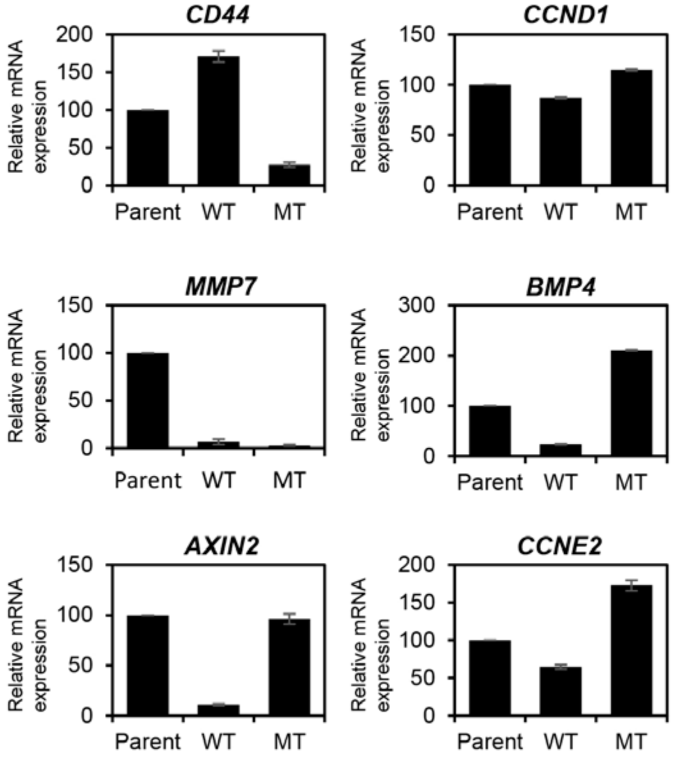

Supplementary Figure S1. qPCR analysis of Wnt/β-catenin signaling target genes, *CD44*, *CCND1*, *MMP7*, *BMP4*, *AXIN2*, and *CCNE2* in HCT116-P, HCT116-WT, and HCT116-MT cells. Error bars represent the standard deviation (SD) of the mean of two independent experiments.

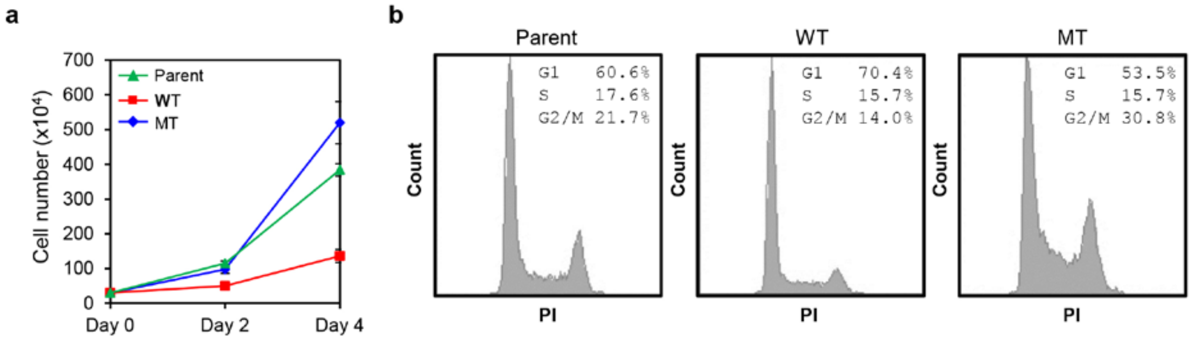

Supplementary Figure S2. β-catenin activation promotes cell cycle upregulation and cell proliferation. **(a)** Cell proliferation assay performed on HCT116 cells containing a wild-type (WT) β-catenin allele (HCT116-WT, WT), a mutant β-catenin allele (HCT116-MT, MT), or both a wild-type and mutant allele (HCT116-P, Parent). Cells were seeded at a density of 3×10^6 and counted manually 2 and 4 days after seeding. **(b)** Propidium iodide flow cytometric cell cycle analysis of all three β-catenin HCT116 cell lines.

a

Major EMT factor (normalized to *GAPDH*)

Gene	F.C. (MT/WT)
TWIST1	1.260202
SNAI1	1.227645
SNAI2	2.114922
ZEB1	2.107306

b

EMT hallmark

Gene	F.C. (MT/WT)
LAMA3	-3.48248
LAMC2	-2.67062
SCG2	-2.35741
RGS4	-2.30539
ID2	-2.27408
MATN2	-1.96483
CD44	-1.90887
EFEMP2	-1.73006
FUCA1	-1.72227
GADD45A	-1.64045
PLAUR	-1.6369
ACTA2	-1.60321
VEGFA	-1.58174
PLOD3	-1.56025
DPYSL3	-1.53545
CYR61	-1.52886
TNFAIP3	1.509536
IL8	1.523821
RHOB	1.543268
FERMT2	1.546939
COL6A3	1.578306
TGM2	1.618568
SPP1	1.698225
DKK1	1.821872
CAP2	2.282853
VCAN	2.330703
MSX1	3.179599
VIM	7.576687

Supplementary Figure S3. Identification of EMT-related genes showing differential expression in HCT116-MT cells, compared to HCT116-WT cells in the microarray data **(a)** The expression of four main EMT factors (*SNAI1*, *SNAI2*, *ZEB1*, and *TWIST1*) was analyzed using microarray data by normalizing them to *GAPDH* expression. **(b)** 1,507 DEGs obtained from microarray data were compared to a set of 200 EMT-related genes obtained from KEGG database. Twenty-eight EMT-related genes were significantly changed in HCT116-MT cells, compared to HCT116-WT cells.

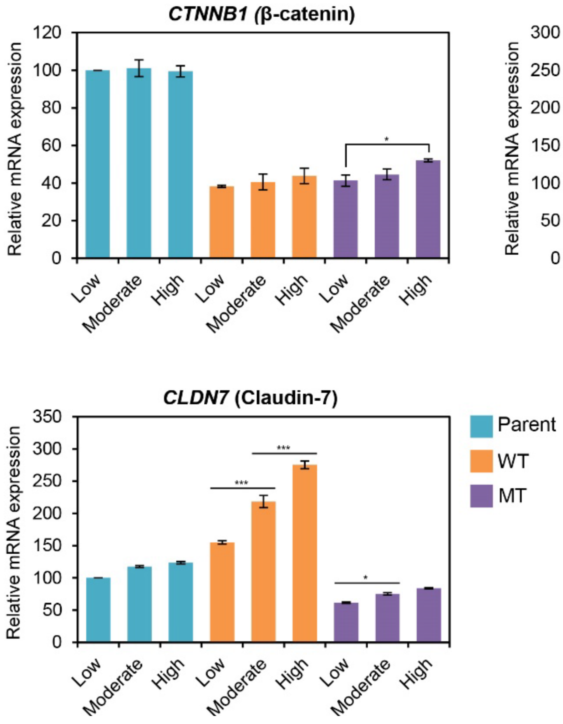

Supplementary Figure S4. HCT116-P, HCT116-MT, and HCT116-WT cells show distinct patterns of cell density-dependent β-catenin, Claudin-7, and E-cadherin mRNA expression. The mRNA expression levels of β-catenin, Claudin-7, and E-cadherin were measured by qRT-PCR in all three β-catenin HCT116 cell lines at low, moderate, and high cell density. Error bars represent the SD of the mean of three independent experiments. One-way analysis of variance (ANOVA) with a post-hoc test (Bonferroni) was performed to compare multiple means. **P* <0.01, ***P* <0.001, ****P* <0.0001. Statistical significance between low (L) and moderate (M) cell densities, and M and high (H) cell densities is shown. Statistical significance between L and H is shown when there is no statistical significance between L and M or M and H.

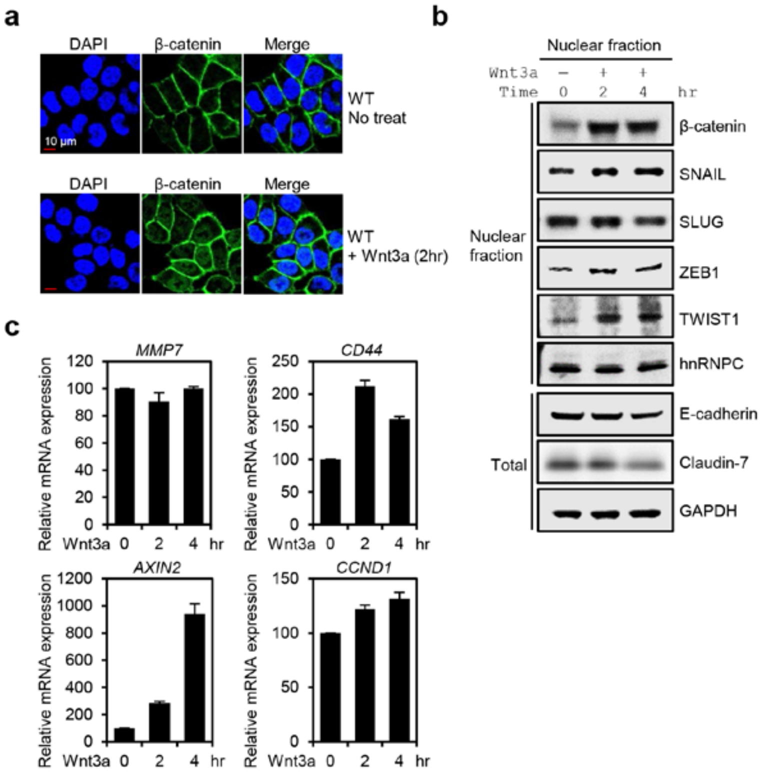

Supplementary Figure S5. Wnt3a treatment induced translocation of β-catenin and EMT transcription factors into the nucleus, as well as loss of E-cadherin and Claudin-7, accompanying transcriptional activation of downstream target genes of Wnt/β-catenin signaling in HCT116-WT cells. **(a)** Immunofluorescence microscopy analysis of HCT116-WT cells with or without 2-hour Wnt3a treatment. **(b)** Western blotting analysis of β-catenin, SNAIL, SLUG, ZEB1, TWIST1, E-cadherin, and Claudin-7 using the nuclear fraction of HCT116-WT cells treated with Wnt3a for 2 and 4 hours. **(c)** qPCR analysis of *MMP7*, *CD44*, *AXIN2*, and *CCND1* in HCT116-WT cells treated with Wnt3a for 2 and 4 hours. Error bars represent the standard deviation (SD) of the mean of two independent experiments.

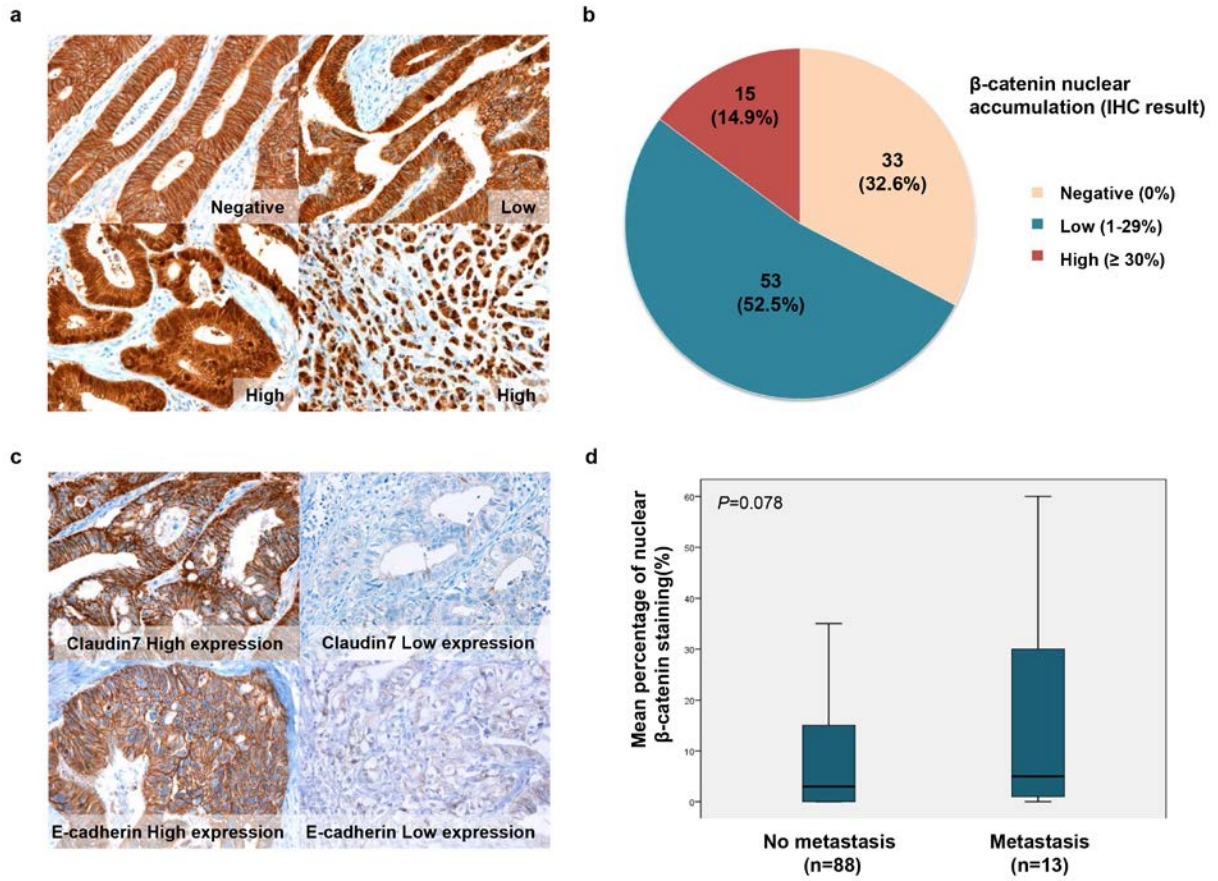

Supplementary Figure S6. Expression of nuclear β-catenin, Claudin-7, and E-cadherin in colorectal cancer (CRC) tissues. **(a)** Representative immunohistochemical analysis of CRC tissues with negative, low, or high nuclear β-catenin expression. Nuclear β-catenin expression was classified as negative (detected 0% of tumor cells), low (detected in 1–29% of tumor cells), or high (detected in ≥30% of tumor cells). **(b)** Proportion of the three nuclear β-catenin expression groups identified in our cohort of 101 CRC tumor samples. **(c)** Representative immunohistochemical analysis of CRC tissues with low or high expression of Claudin-7 and E cadherin. If the H-score (see Materials and Methods) of Claudin-7 or E cadherin was >200, the sample was classified as high expression (left, upper and lower), and if the H-score ≤200, the sample was classified as low expression (right, upper and lower). **(d)** The mean percentage of nuclear β-catenin-staining in CRC samples that displayed metastasis after operation and in those that did not.

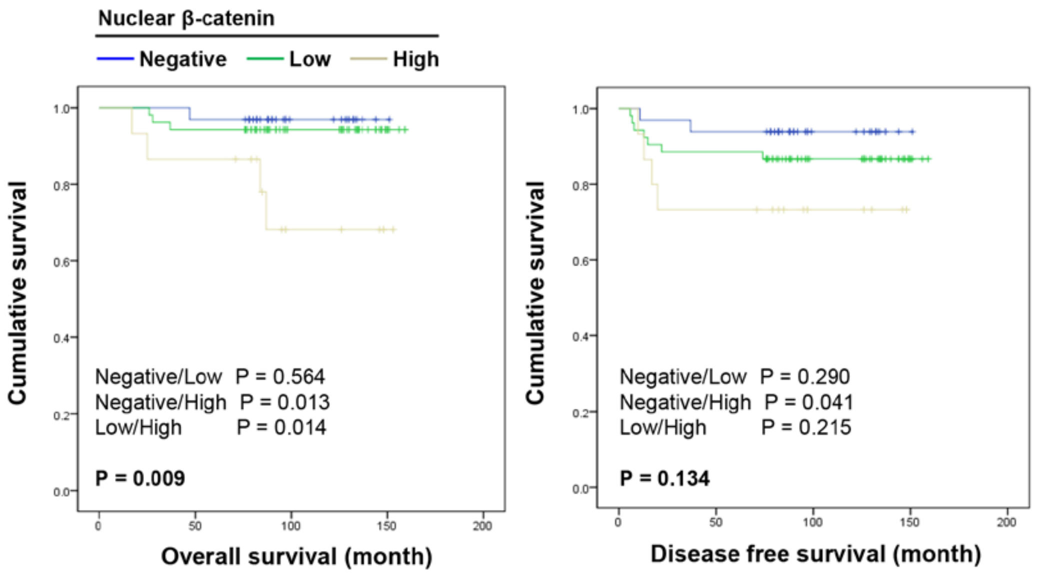

Supplementary Figure S7. Kaplan–Meier analysis of 101 CRC patients according to nuclear β-catenin expression. Using 101 CRCs, cases with no nuclear β-catenin expression (n = 33), cases with low nuclear β-catenin expression (n = 53), and cases with high nuclear β-catenin expression (n = 15) were subjected to the overall survival (P = 0.009) and disease-free survival (P = 0.134) analysis.

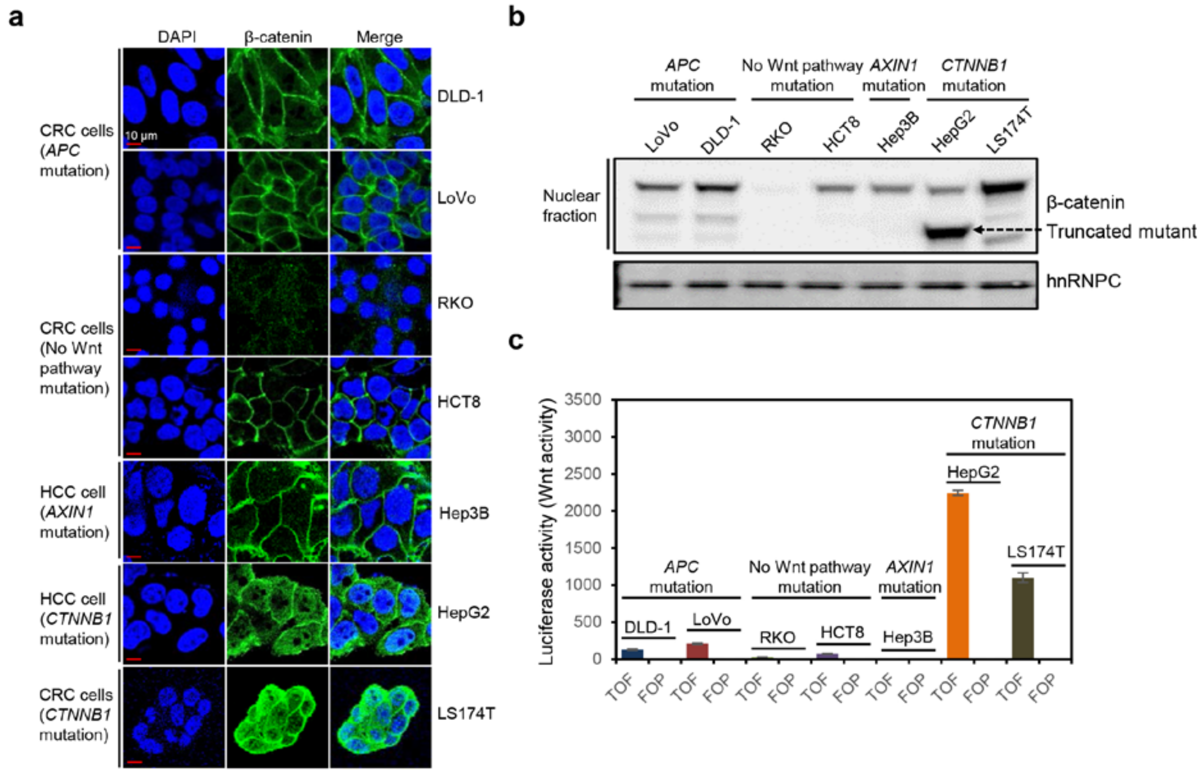

Supplementary Figure S8. Localization and nuclear expression of β-catenin, and Wnt pathway activity in cell lines with Wnt pathway related mutations or not. **(a,b)** Immunofluorescence microscopy and western blotting analysis of β-catenin in two APC mutant CRC cell lines (DLD-1 and LoVo), CRC cell lines with no Wnt pathway related mutations (RKO and HCT8), a hepatocellular carcinoma (HCC) cell line with *AXIN1* mutation (Hep3B), a HCC cell line with *CTNNB1* mutation (HepG2), and a CRC cell line with *CTNNB1* mutation (LS174T). Nuclear fraction of cell lysates was used for western blotting analysis. Black arrow indicates mutant β-catenin. **(c)** WNT pathway activity was measured by TOP/FOP luciferase reporter assay. Error bars represent the standard deviation (SD) of the mean of two independent experiments.

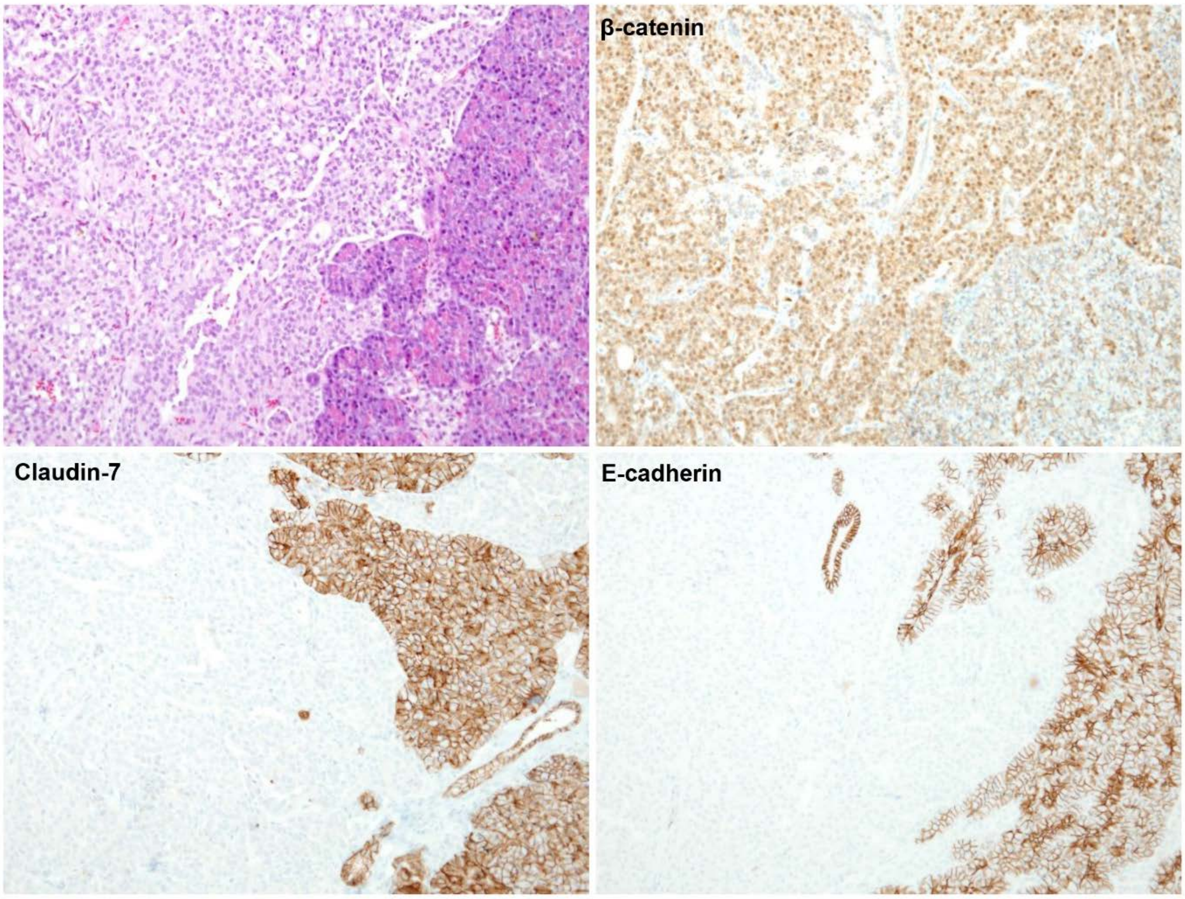

Supplementary Figure S9. Solid-pseudopapillary neoplasm (SPN) tissues show specific and strong nuclear β-catenin expression, accompanied by complete loss of both E-cadherin and Claudin-7 expression. Expression of β-catenin, E-cadherin, and Claudin-7 was measured by immunohistochemistry in 15 SPN samples. Representative protein-specific and hematoxylin and eosin staining (H&E) results are shown.

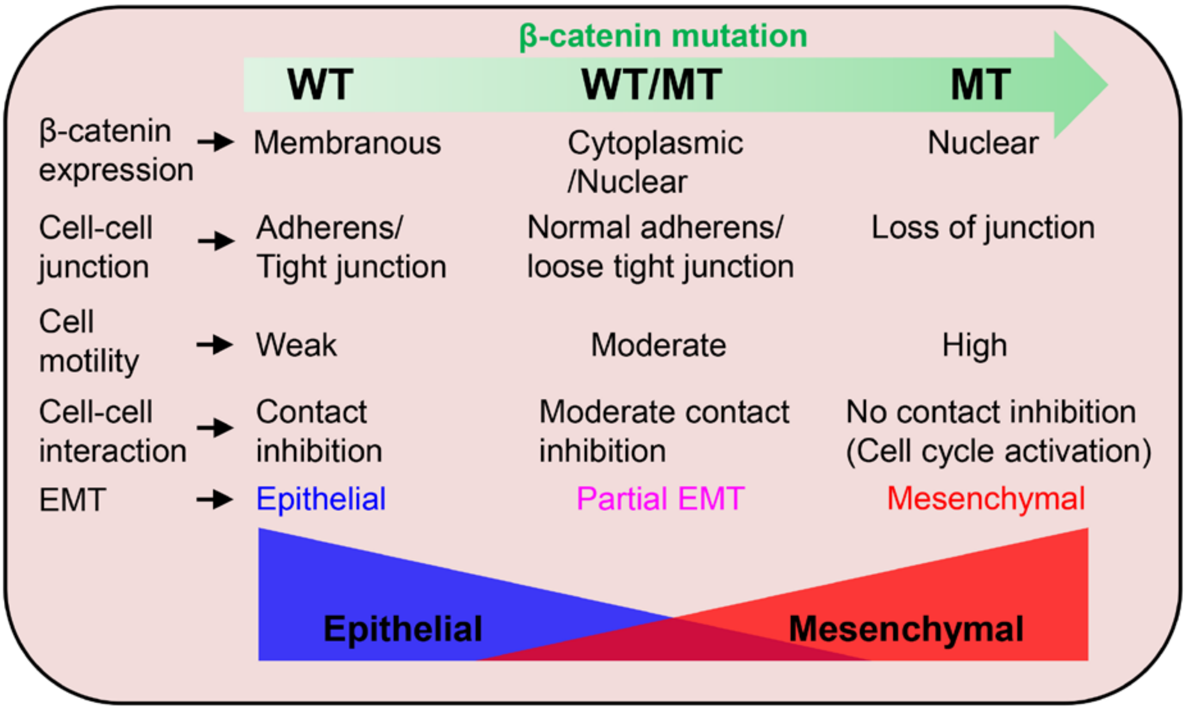

Supplementary Figure S10. Model describing the relationship between β-catenin activation status and EMT progression in CRCs.

Supplementary Table S1. siRNA sequences against the four EMT factors

Gene	siRNA sequence
SNAI1	5'- GAUGCAGUUCCGCUCCAUU d(UU)-3’
SNAI2	5'- GAGCAAUCAAGCAGAUCAU d(UU)-3’
ZEB1	5'- CGCUCUGUUGAAGGCUGGA d(UU)-3’
TWIST1	5'- GTGTATGTGCGCCAAAGTA d(UU)-3’

Supplementary Table S2. Pathway analysis of up- or down-regulated genes (|fold change|>1.5) in HCT116-MT cells compared to HCT116-WT cells

Pathway analysis of down-regulated genes			
Category	Term	P-Value	Benjamini
KEGG_PATHWAY	Antigen processing and presentation	5.00E-09	7.80E-07
KEGG_PATHWAY	Cell adhesion molecules (CAMs)	1.00E-05	7.90E-04
KEGG_PATHWAY	Type I diabetes mellitus	1.70E-05	8.80E-04
KEGG_PATHWAY	Allograft rejection	3.00E-05	1.20E-03
KEGG_PATHWAY	Graft-versus-host disease	5.90E-05	1.80E-03
KEGG_PATHWAY	Lysosome	9.80E-05	2.50E-03
KEGG_PATHWAY	Viral myocarditis	4.10E-04	9.10E-03
KEGG_PATHWAY	Autoimmune thyroid disease	5.20E-04	1.00E-02
KEGG_PATHWAY	Systemic lupus erythematosus	6.60E-04	1.10E-02
KEGG_PATHWAY	Other glycan degradation	5.50E-03	8.20E-02
KEGG_PATHWAY	Small cell lung cancer	4.30E-02	4.60E-01
KEGG_PATHWAY	ECM-receptor interaction	4.30E-02	4.60E-01
KEGG_PATHWAY	Asthma	4.50E-02	4.50E-01
KEGG_PATHWAY	Tight junction	4.90E-02	4.50E-01
Pathway analysis of up-regulated genes			
Category	Term	P-Value	Benjamini
KEGG_PATHWAY	Ribosome	9.20E-06	1.30E-03
KEGG_PATHWAY	Spliceosome	1.50E-03	1.10E-01
KEGG_PATHWAY	Cell cycle	4.50E-03	1.90E-01
KEGG_PATHWAY	Purine metabolism	7.60E-03	2.40E-01
KEGG_PATHWAY	DNA replication	8.10E-03	2.10E-01
KEGG_PATHWAY	Limonene and pinene degradation	1.20E-02	2.50E-01
KEGG_PATHWAY	Pancreatic cancer	1.30E-02	2.40E-01
KEGG_PATHWAY	Pyrimidine metabolism	1.90E-02	2.90E-01
KEGG_PATHWAY	Proteasome	2.40E-02	3.30E-01
KEGG_PATHWAY	Fatty acid elongation in mitochondria	3.00E-02	3.60E-01

Supplementary Table S3. The clinicopathologic features of 101 CRCs according to nuclear β-catenin expression status

		β-catenin nuclear accumulation						Total	p-value
		Absent		Low		High			
		n = 33, 32.6%		n = 53, 52.5%		n = 15, 14.9%			
**Age**		60.18±11.15		57.64±11.13		61.07±11.52		101	0.449
**Sex**	Male	**16**	48.5%	**27**	50.9%	**8**	53.3%	51	0.948
	Female	**17**	51.5%	**26**	49.1%	**7**	46.7%	50	
**Location**	Right side	**17**	51.5%	**14**	26.4%	**5**	33.3%	36	0.060
	Left side	**16**	48.5%	**39**	73.6%	**10**	66.7%	65	
**MSI**	MSS and MSI low	**27**	81.8%	**52**	98.1%	**14**	93.3%	93	0.013
	MSI high	**6**	18.2%	**1**	1.9%	**1**	6.7%	8	
**Pre-operative CEA level**	≤5ng/ml	**27**	81.8%	**35**	66.0%	**11**	73.3%	73	0.281
	>5ng/ml	**6**	18.2%	**18**	34.0%	**4**	26.7%	28	
**Gross type**	Exophytic	**28**	84.8%	**38**	71.7%	**11**	73.3%	77	0.364
	Non exophytic	**5**	15.2%	**15**	28.3%	**4**	26.7%	24	
**Size**	<5cm	**12**	36.4%	**33**	62.3%	**5**	33.3%	50	0.026
	≥5cm	**21**	63.6%	**20**	37.7%	**10**	66.7%	51	
**Pathologic diagnosis**	Adenocarcinoma	**31**	93.9%	**51**	96.2%	**13**	86.7%	95	0.288
	Mucinous adenocarcinoma	**1**	3.0%	**1**	1.9%	**2**	13.3%	4	
	Medullary carcinoma	**1**	3.0%	**1**	1.9%	**0**	0.0%	2	
**Metastasis after operation**	Absent	**31**	93.9%	**46**	86.8%	**11**	73.3%	88	0.142
	Present	**2**	6.1%	**7**	13.2%	**4**	26.7%	13	
**E-cadherin**	High expression	**25**	75.8%	**39**	73.6%	**7**	46.7%	71	0.093
	Low expression	**8**	24.2%	**14**	26.4%	**8**	53.3%	30	
**Claudin7**	High expression	**20**	60.6%	**34**	64.2%	**7**	46.7%	61	0.474
	Low expression	**13**	39.4%	**19**	35.8%	**8**	53.3%	40	

Supplementary Information (Blots)

Fig. 1C

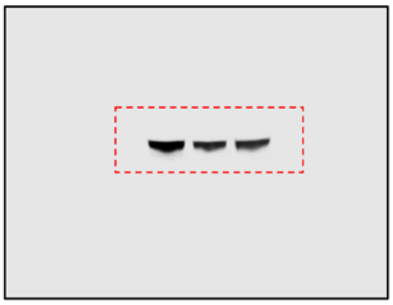

β-catenin

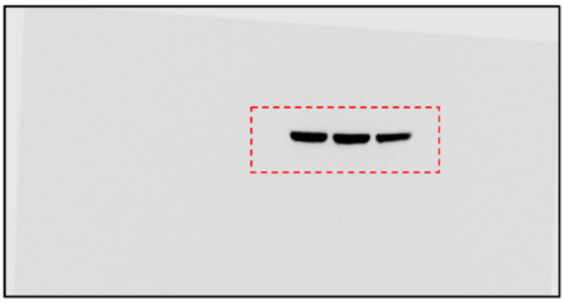

GAPDH

Fig. 1E

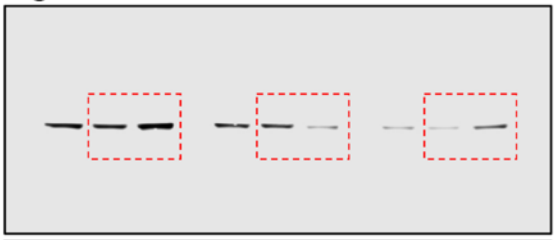

β-catenin

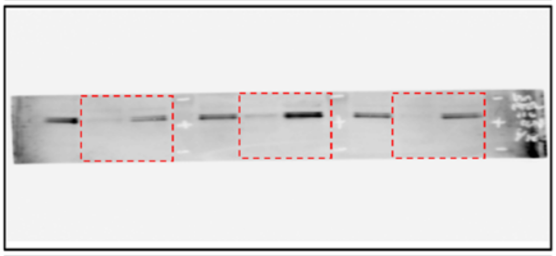

hnRNPC

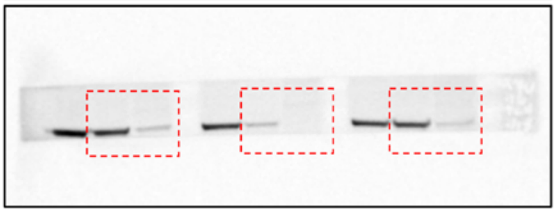

α-tubulin

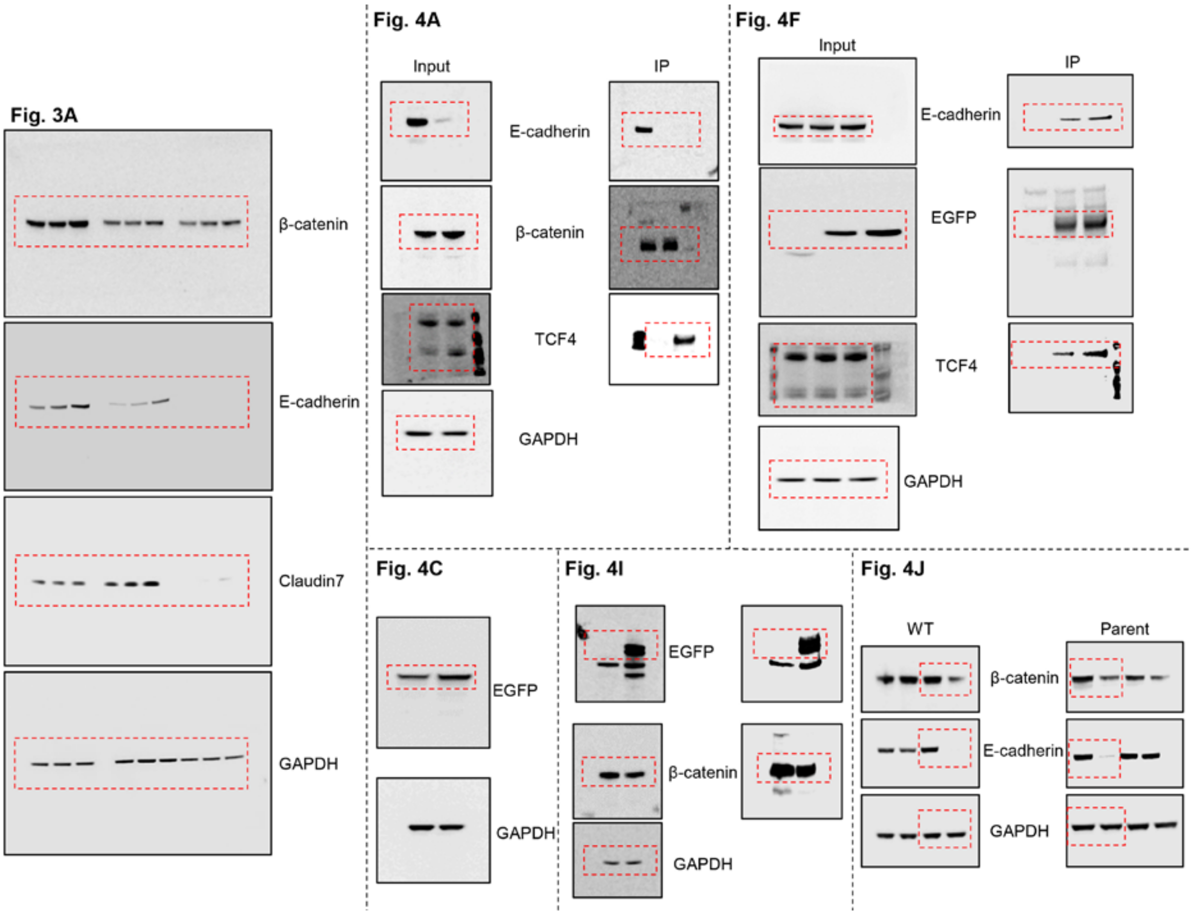

Fig. 3A
β-catenin
E-cadherin
Claudin7
GAPDH
Fig. 4A
Input
IP
E-cadherin
β-catenin
TCF4
GAPDH
Fig. 4F
Input
IP
E-cadherin
EGFP
TCF4
GAPDH
Fig. 4C
EGFP
GAPDH
Fig. 4I
EGFP
β-catenin
GAPDH
Fig. 4J
WT
Parent
β-catenin
E-cadherin
GAPDH

Fig. 6B

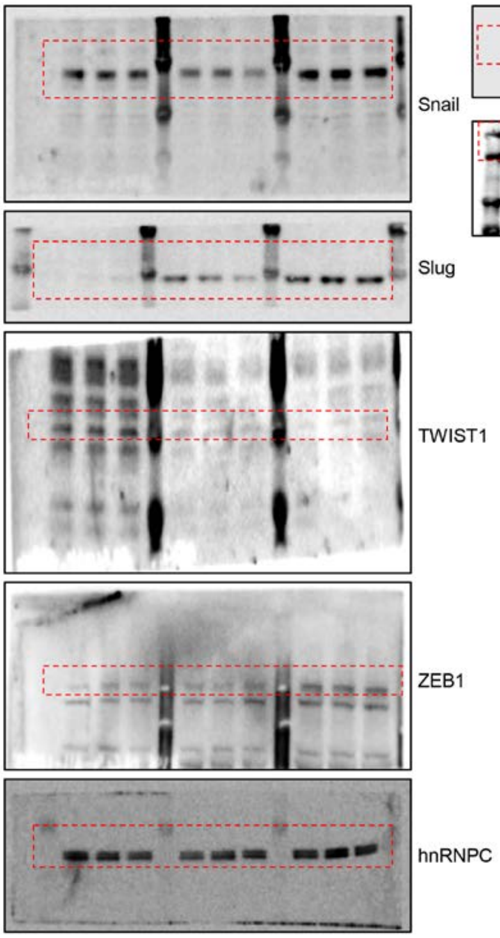

Snail
Slug
TWIST1
ZEB1
hnRNPC

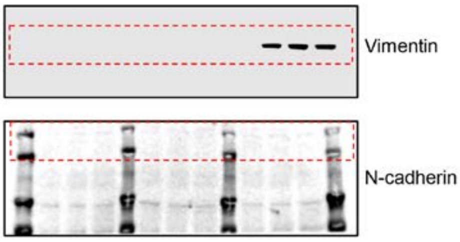

Vimentin
N-cadherin

Fig. 6E

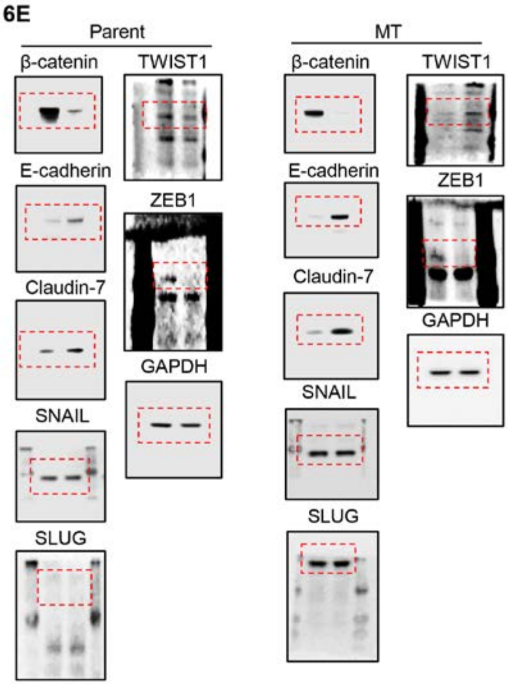

Parent
MT
β-catenin
TWIST1
E-cadherin
ZEB1
Claudin-7
GAPDH
SNAIL
SLUG

Fig. 6F

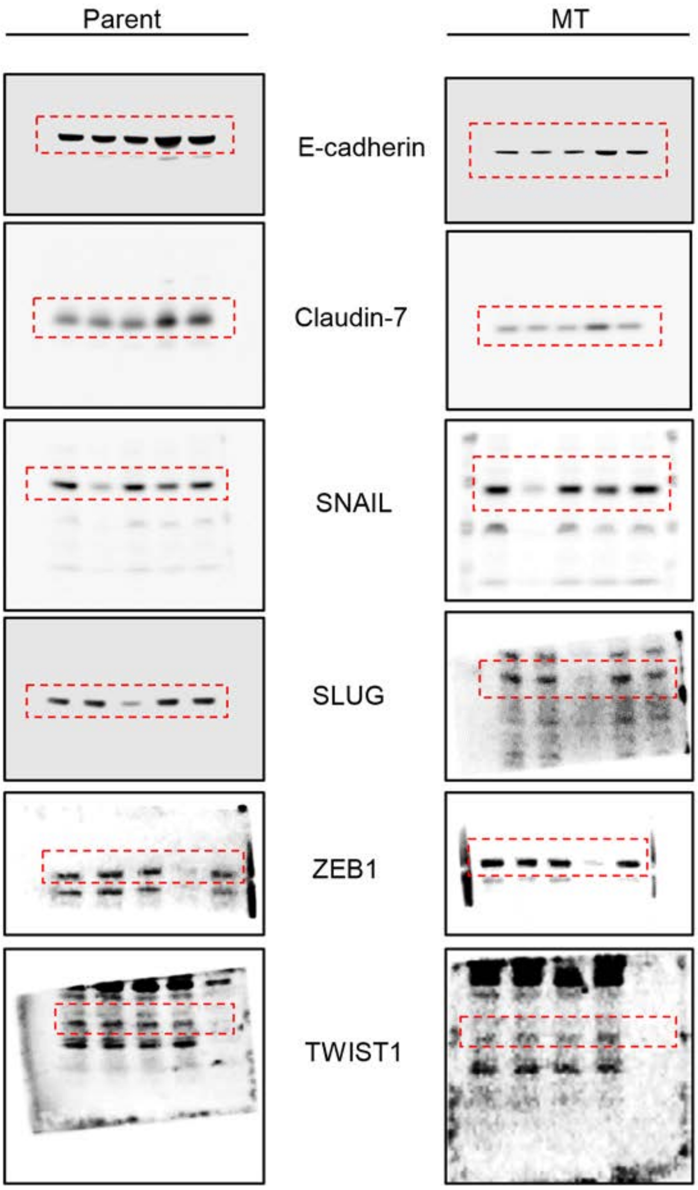

Fig. 7A

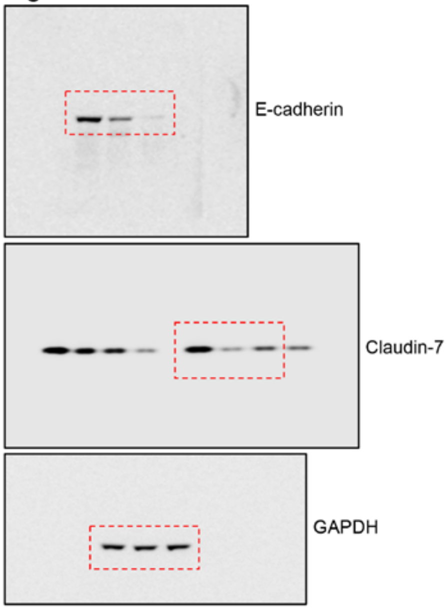

Fig. 8A

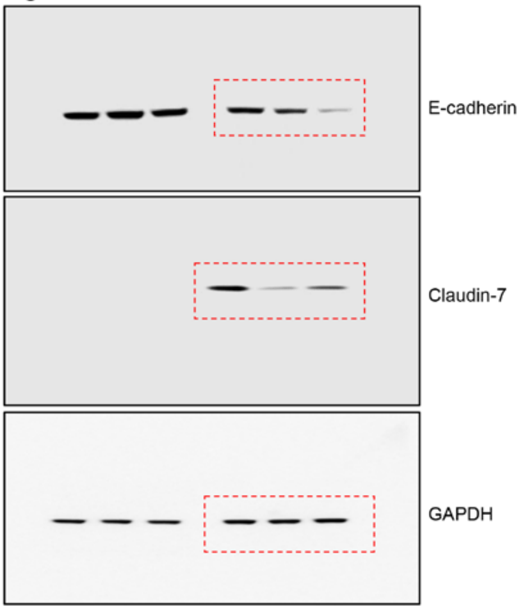

Supplementary Fig. 5B

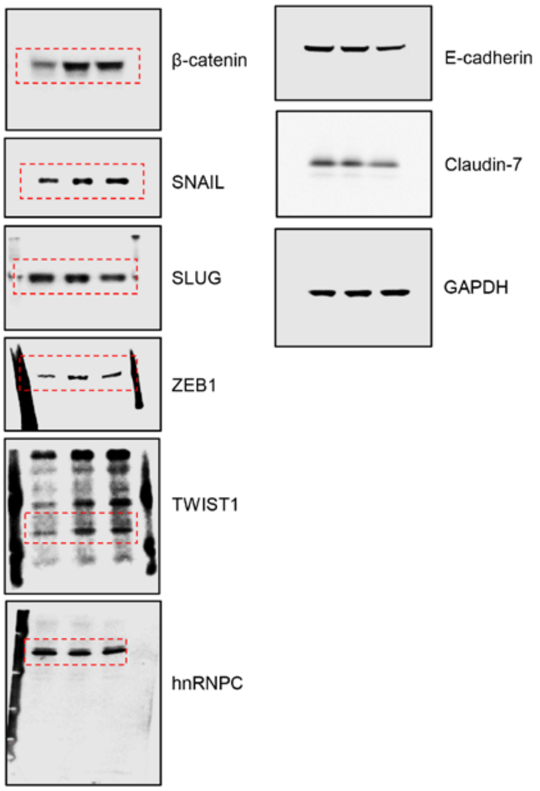

Supplementary Fig. 8

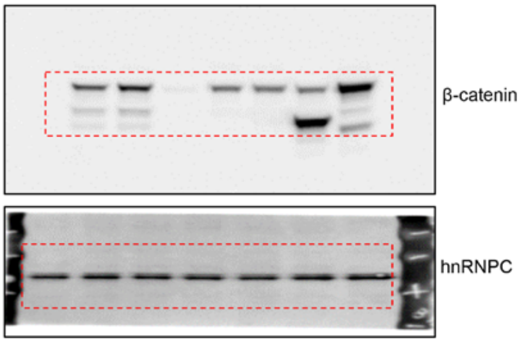

Supplement: Supplementary file 1 — Supplementary Information [file 41598_2019_54890_MOESM1_ESM.pdf]
